# HT-SuMD: making molecular dynamics simulations suitable for fragment-based screening. A comparative study with NMR

**DOI:** 10.1080/14756366.2020.1838499

**Published:** 2020-10-28

**Authors:** Francesca Ferrari, Maicol Bissaro, Simone Fabbian, Jessica De Almeida Roger, Stefano Mammi, Stefano Moro, Massimo Bellanda, Mattia Sturlese

**Affiliations:** a Department of Chemical Sciences, University of Padova, Padova, Italy; b Molecular Modeling Section (MMS), Department of Pharmaceutical and Pharmacological Sciences, University of Padova, Padova, Italy

**Keywords:** FBLD, MD, NMR, Bcl-XL, SuMD

## Abstract

Fragment-based lead discovery (FBLD) is one of the most efficient methods to develop new drugs. We present here a new computational protocol called High-Throughput Supervised Molecular Dynamics (HT-SuMD), which makes it possible to automatically screen up to thousands of fragments, representing therefore a new valuable resource to prioritise fragments in FBLD campaigns. The protocol was applied to Bcl-X_L_, an oncological protein target involved in the regulation of apoptosis through protein–protein interactions. Initially, HT-SuMD performances were validated against a robust NMR-based screening, using the same set of 100 fragments. These independent results showed a remarkable agreement between the two methods. Then, a virtual screening on a larger library of additional 300 fragments was carried out and the best hits were validated by NMR. Remarkably, all the in silico selected fragments were confirmed as Bcl-X_L_ binders. This represents, to date, the largest computational fragments screening entirely based on MD.

## 1. Introduction

Since its introduction over 20 years ago, the technique of Fragment-based lead discovery (FBLD) has turned out to be one of the most effective methods in the development of new drugs. Nowadays, a remarkable number of companies and university laboratories adopt this approach in their pipeline, as demonstrated by the presence of at least 30 FBLD-derived molecules in various phases of clinical development^1^. The lower functionalisation and chemical complexity typical of fragments (usually having less than 20 heavy atoms) result in weak interactions with the biological target and thus in modest binding affinities, that span the range from mM to high µM. Different from classical HTS, the aim of FBLD is the identification of weak binding fragments, starting from which, through an optimisation procedure, it is possible to develop a mature candidate^2^. The screening of small fragments, in comparison to traditional HTS screening, has several and notable advantages: (i) it facilitates larger sampling of chemical space; (ii) FBLD has a higher hit rate;(iii) the lead compounds obtained from fragment-based campaigns have better ligand efficiency (LE) and they are more hydrophilic^2–4^. Sensitive screening technologies are essential to detect the weak interactions mediated by fragments: NMR spectroscopy, isothermal titration calorimetry (ITC), differential scanning fluorimetry (thermal shift), surface plasmon resonance (SPR), and X-ray crystallography represent robust approaches for fragment screening. Protein-based NMR and X-ray crystallography are widely adopted because they can provide detailed information on the molecules binding mode. Structural data are a valuable help in fragment maturation, but they are suitable only for certain targets and they still require a very long time to be collected and analysed, thus representing a bottleneck in drug discovery campaigns. These limitations have made the integration of computational methods for fragment screening increasingly attractive. For instance, Molecular docking can be exploited to evaluate in silico, within a relatively short time, up to millions of fragments towards one specific target. Although several examples of docking applications to FBLD are described in the literature, its routine use remains challenging: the majority of scoring functions are trained on mature compounds, which often renders them inadequate to distinguish true binding fragments from false positives^5–7^. Computational protocols such as Multiple-Copy Simultaneous Search (MCSS), providing an interactive map of a protein binding site through the iterative reorientation of small functional groups, have also gained increasing importance in the field^8^ . The integration of molecular dynamics simulations (MD) with FBLD is however more appealing since it would allow a better investigation of molecular recognition, taking also into account the flexibility of the protein target along time. Unfortunately, binding is a rare event and its sampling using unbiased MD techniques requires long-timescale simulations, making the approach difficult to reconcile with HTS. A first pioneering attempt to overcome these issues was performed using an adaptive MD simulations scheme: six months were necessary to perform the screening of about one hundred fragments (for a total of 5.85 ms) of simulations, using a massive GPU cluster^9^. Although the results collected were encouraging, the time required for the in silico simulations was not very competitive, if compared to the classic experimental approaches. Similarly, in a recent investigation, long µs timescale MD simulations were exploited for the prediction of fragments binding mode by Markov-state models (MSMs). Also in this case, the convergence of the results was bound to an extensive computation effort, making the technique scarcely prone to large-scale implementation^10^.

In our laboratory, a methodology called supervised molecular dynamics (SuMD) was recently developed. It is able to accelerate the sampling of binding events to a nanosecond timescale, without introducing any energetic bias to the simulation^11–13^. First attempts in investigating the recognition of fragments with a weak affinity (K_d_ in the milli- to the micromolar range) showed that SuMD was able to reproduce the final bound-state reported in experimentally solved structures with root mean square deviations (RMSD) even below 1 Å^12^. Most interestingly, this technique allows the ligand to dynamically explore the ligand-binding site. This aspect is particularly relevant for weak ligands that are more prone to fluctuate in the binding site, showing multiple binding modes, rather than settle in a univocal conformation.

Here, we present for the first time the implementation of SuMD in a High Throughput computational platform (HT-SuMD), capable to screen thousands of fragments even in a small GPU cluster. To test the performance in a real fragment screening campaign, we carried out a parallel screening comparing the outcome of HT-SuMD with a robust NMR-based experimental protocol. The antiapoptotic member of the Bcl-2 family, Bcl-X_L_, was selected as a case study. Bcl-X_L_ is a protein suitable for NMR screening and exemplary in FBLD because of its most well-known inhibitor ABT-737, which was developed using a fragment-based approach^14^. The Bcl-X_L_ binding cleft accommodates the long helical domain of BH3 proapoptotic proteins and, as a consequence, its vast site is shaped by contiguous hydrophobic pockets (pocket P1 to P4)^15^. One of the major challenges in Bcl-X_L_ targeting is represented by the flexibility of the helices surrounding the binding domain, specifically helix-3, which can adopt different conformations depending on the ligand hosted in the cleft^16^^,^^17^. The flexibility of the binding site is challenging for those in silico structure-based strategies, such as molecular docking, that cannot take into account such significant target plasticity. All these reasons make Bcl-X_L_ an attractive system to challenge HT-SuMD performance, comparing the in silico outcomes with NMR. It is worth remembering that this target is not suitable for X-ray soaking fragment screening, a fact that further limits the FBLD approach^18^^,^^19^.

## 2. Materials and methods

### 2.1. Molecular modelling: software and hardware overview

The MOE suite (Molecular Operating Environment, version 2019.0101) was exploited to perform most general molecular modelling operations, such as protein and ligands preparation^20^. All these operations were performed on an 8 CPU (Intel® Xeon® CPU E5-1620 3.50 GHz) Linux workstation. In silico screening of fragments was performed in an automated fashion by HT-SuMD, a hybrid in house code, written in Python, Bash, and Tcl languages. Molecular dynamics simulations were performed employing the ACEMD engine on a GPU heterogeneous cluster composed of 20 NVIDIA GPUs (models spanning from GTX 780 to Titan Xp). The combination AMBER 14SB/general Amber force field (GAFF) was adopted for all MD simulations^21^^,^^22^. Trajectories analysis was also performed by HT-SuMD in a 64 core CPU cluster (AMD Opteron™ CPU 6376 2.30 GHz).

### 2.2. Systems preparation

#### 2.2.1. Three-dimensional structure of Bcl-X_L_

At present, the RCSB Protein Data Bank database (PDB) stores 75 three-dimensional structures of the human protein Bcl-X_L_, identifiable by the UniProtKB accession code Q07817^23^ . Among the many entries available, solved with different experimental techniques and in the presence of different ligands, we chose for this study the NMR structure PDB-ID 1G5J^24^ as this structure ensures the maximum closeness in terms of sequence identity between the engineered protein expressed and exploited in NMR experiments and the one computationally used, thus ensuring that the simulated dynamics correspond to that of the experimentally used protein construct.

Coordinates of the complex were downloaded and processed with the protein preparation tool as implemented in MOE^20^. The appropriate ionisation states (at pH 7.4) of titratable residues were established using the Protonate-3D tool. Only the protein chain corresponding to Bcl-X_L_ was included in the preparation protocol. The C-terminus was capped to mimic the missing residues^25^.

#### 2.2.2. Fragments library

A multipurpose in-house library composed of 400 fragments was used for both screenings. It was set up by cherry-picking fragments from high quality commercially available libraries of different vendors (LifeChemicals, Sigma-Aldrich, OTAVAchemicals; the fragments purity was over 95%). Moreover, all the fragments were proved to be soluble at 100 mM in deuterated DMSO and at least 1 mM in phosphate buffer. The three-dimensional structures of the fragments were built taking advantage of the MOE-Builder tool and the ionisation states at physiological conditions were predicted using the MOE.^20^ Fragment structures were subjected to MMFF94× energy minimisation until the root mean square (RMS) gradient fell below 0.05 kcal mol^−1^ Å ^−1^.

### 2.3. Ht-SuMD protocol

HT-SuMD is a platform written in Python, Bash, SVL, and Tcl that enclose s SuMD code with a set of tools devoted to MD system preparation and the analysis of recognition trajectories analysis.

HT-SuMD allows us to prepare, run, and analyse thousands of SuMD simulations in a fully automated fashion^11^. The only inputs required by the HT-SuMD protocol are the 3 D structures of the protein target (PDB file format) and a molecular database containing the fragments that have to be investigated (sd file format). Below, the peculiar steps underlying HT-SuMD functionality are described. SuMD allows exploration of the entire ligand–receptor recognition pathway, from the unbound to the bound state by collecting short unbiased MD simulations and monitoring how the protein–ligand distance varies over time. Briefly, a tabu-like algorithm at the of each short MD simulation (named SuMD step) accepts and prosecutes all the productive SuMD steps in which an approach of the ligand is sampled, otherwise, it rejects and simulates again from the previous coordinates set those steps describing instead a diffusion of the ligand far from the target. The combination of the accepted SuMD steps results in the SuMD trajectory.

#### 2.3.1. Sumd systems setup

HT-SuMD generates as many molecular dynamics systems as the number of ligands contained in the fragments database. Each system is composed of a macromolecular target protein (in our case, Bcl-X_L_), and a single fragment molecule. To avoid protein–ligand long-range interactions in the starting geometry, all ligands were positioned at the origin of the cartesian system, far from the protein binding site, at a distance bigger than the electrostatic cut-off term used in the simulation (9 Å). T-Leap, part of the ambertools2014 suite^26^, was exploited for the generation of protein–ligand complexes, using AMBER14SB as a force field for the protein^21^. The parameters necessary to describe and simulate fragments were instead retrieved in the GAFF^22^^,^^27^, by using the antechamber and parmchk tools^21^^,^^22^. After geometry minimisation, ligand partial charges were calculated following the procedure suggested by antechamber, with semiempirical method AM1-BCC^28^^,^^29^. Each system was explicitly solvated using TIP3P as water model in a cubic box with borders placed at least 13 Å away from any protein or ligand atom. The total charge of each box was neutralised adding a specific number of Na^+^/Cl^−^ counterions, to ensure a final salt concentration of 0.1 M. All the systems were energy minimised performing 600 steps with the conjugate-gradient method, then 50000 steps of NVT (0.1 ns) followed by 400000 steps of NPT simulations (0.8 ns). Both equilibration simulations were carried out using a time step of 2 fs and applying harmonic positional constraints on protein and fragment heavy atoms with a force constant of 1 kcal mol^−1 ^Å ^−2^, gradually reduced with a scaling factor of 0.1. The temperature was maintained at 310 K by a Langevin thermostat and in the NPT simulation, the pressure was maintained at 1 atm by a Berendsen barostat^30^.

#### 2.3.2. Sumd simulations collection

Once all the systems have been prepared and equilibrated, HT-SuMD controls the collection of SuMD trajectories in an automated manner, partitioning the simulations depending on GPUs’ cluster dimension. Similar to the original implementation, also in this protocol the supervision algorithm of SuMD monitors in time the distance between the fragments mass centres and the target binding site (d_cm(F-T)_), accelerating the sampling of binding events^11–13^. A tabu like algorithm evaluates Since small fragments are characterised by a more pronounced diffusive motion, if compared to mature compounds or peptides, it has been possible to shrink the length of each suMD steps from the original value of 600 ps to 300 ps, further increasing protocol performance. For each simulation box and, thus, for each fragment, three different replicas were produced, to improve the accuracy of the simulations.

#### 2.3.3. Sumd trajectories analysis

Each SuMD trajectories produced was geometrically analysed to identify significant populations of ligand conformations, among the multitude of sampled data. Prody, a python framework for MD manipulation and analysis, was exploited to compute the pairwise RMSDs of fragment atomic coordinates, during the different simulations^31^. From each replica, a square matrix of RMSDs was obtained (n_f_ x n_f_), in which n_f_ is the number of frames, and thus of ligand conformations, characterising that specific SuMD binding trajectory. Subsequently, DBSCAN, a density-based clustering algorithm part of the scikit-learn python packages, was applied to cluster the different fragments conformations^32^^,^^33^. The only parameters necessary for clustering are min_samples, i.e. the minimum number of frames required to initialise a cluster and eps, or the geometric discriminant that determines whether or not two conformations belong to the same cluster. All SuMD trajectories were then clustered using a value of ε equal to 1.5 and a minimum dimension of 10 conformations, resulting in 4268 clusters of fragments conformations, starting from 1200 trajectories, which represented the starting point for all subsequent analysis. The best conformer was extracted based on the best interaction energy.

##### 2.3.3.1 Clusters population analysis

The value of the population density was calculated and expressed both as the absolute number of frames and as a percentage value, calculated with respect to the total number of SuMD frames belonging to the trajectory from which the cluster was generated.

##### 2.3.3.2 Hydrogen bond s analysis

Each cluster of conformations was then analysed to map the presence of HBonds between the different fragments and the Bcl-X_L_ binding site. The analysis was performed through a Tcl script exploiting the HBonds Plugin (v. 1.2), as implemented in the VMD software^34^. The geometrical criteria chosen to identify HBonds were a distance between the donor and acceptor heavy atom lower than 3.0 Å and the angle between donor heavy atom, hydrogen and the acceptor heavy atom higher than 120°. When the criteria were met, the HBonds was recorded, also indicating the nature of the binding site residue involved and the percentage of frames with respect to the total size of the cluster in which the interaction was present.

##### 2.3.3.3 Hydrophobic contribution analysis

To compute a score considering the hydrophobic contribution to binding (HYD) a script based on Scientific Vector Language (SVL) implemented in the MOE suite was used^20^. HYD is an adimensional score (the higher the better). It was calculated for each fragment conformation belonging to a cluster. Finally, the average value HYD_ave_ and the best score HYD_best_ was obtained for each cluster.

##### 2.3.3.4. Energetic analysis

To monitor the strength of the fragment–protein interaction, the ligand-binding affinity (ΔG_bind_) for each clustered conformation was roughly predicted using the MM/GBSA scheme as implemented in AmberTools2014. The complex conformation having the best value (ΔG_best_) was selected as representative of the cluster. Finally, the average ΔG_bind_ (ΔG_ave_) for each cluster was calculated to compare clusters.

#### 2.3.4. Cluster ranking

A consensus scoring approach was then developed to sort the remaining group of fragment conformations. For each of the three observables considered, i.e. mean MMGBSA value (MMGBSA_clust_), mean hydrophobic contribution (HYD_clust_), and clusters size (SIZE_clust_), three independent ranks were first built and then filtered, keeping only those clusters that fit in the top 10% positions of the respective charts. Components of each rank were then compared using a python script, to identify the clusters showing convergence between the different scores and the Venn diagram was produced^35^. Finally, only the highest-score cluster for each ligand was retained.

### 2.4. Protein expression and purification

The human Bcl-X_L_ lacking the C-terminus domain (Δ209-233) was expressed as a 6His-tagged protein in *Escherichia coli* strain BL21(DE3). Transformed bacteria were grown at 37 °C, 220 rpm up to an Optical Density (OD_600 nm_) of 0.6–0.8, and protein expression induced overnight at 20 °C, 220 rpm with 1 mM IPTG. The unlabelled protein was expressed in LB medium while 15 N-labelled protein was produced in M9 minimal medium supplemented with 1 g/L 15NH4Cl and, 4 g/L unlabelled glucose. Ampicillin (100 µg/mL) was added as a selection agent. Cells were harvested by centrifugation (5000 *g*, 20 min, 4 °C) and resuspended in 20 mM PO4 (pH 8.0), 500 mM NaCl, 15 mM imidazole, and 10 mM β-mercaptoethanol, supplemented by Roche cOmplete protease inhibitors cocktail. The 6xHis-tagged Bcl-X_L_ was purified by Ni^2+^-affinity chromatography on a HisTrap FF crude column (GE Healthcare). To ensure the high final purity of Bcl-X_L_, a further step of purification was carried out by size exclusion chromatography (SEC) on a Superdex G-75 column (GE Healthcare), equilibrated within 20 mM TRIS (pH 7.4), 150 mM NaCl and 1 mM DTT.

### 2.5. NMR-based screening

All the NMR screening experiments were acquired with a Bruker DMX 600 MHz spectrometer, equipped with a 5 mm room temperature TXI probe, at 298 K. The initial screening of 100 fragments was conducted with a protein-based approach, by acquiring ^15 ^N SOFAST-HMQC^36^ spectra on 80 µM ^15 ^N-labelled Bcl-X_L_ dissolved in 20 mM TRIS, 150 mM NaCl, 1 mM DTT, pH 7.4, in the presence or in the absence of added compounds. The SOFAST-HMQC experiments were acquired with 48 scans, a recovery delay of 200 ms before each scan, and 120 increments in the indirect dimension for a total acquisition time of around 27 min. The number of points in the indirect dimension was doubled by linear prediction in the processing scheme. The screening was performed adding to the protein 5 compounds, at a concentration at 640 μM each, for a total of 20 mixtures, These mixtures were created with the program NMRmix^37^ to minimise overlap in the 1 D ^1^H NMR spectra and to facilitate therefore the mixture deconvolution performed, in the second step of our protocol, with a ligand-based approach. The percentage of DMSO_d_6_ in every tube was 3%, an amount that did not induce significant chemical shift perturbation on Bcl-X_L_. The pH of all NMR samples was carefully adjusted at 7.4 ± 0.05 before each measurement. The 2 D spectra of the mixtures were analysed using a selection of 19 peaks chosen according to the following criteria. First, peaks had to be a probe for the specific binding with the protein, so the selected peaks had to refer to residues in the protein binding cavity or in its proximity. Second, only isolated or partially overlapped peaks were chosen, so that they could be unambiguously followed during the titration.

Resonance assignment was achieved by comparison with data available in the BMRB database^38^ (BMRB accession numbers 30150, 6578, 25466, and 36133). The 19 selected peaks are E92, A93, G94, D95, E98, L99, R102, A104, L108, S110, Q111, L112, E129, D133, G134, G138, A142, F146, G196. For these 19 peaks, the chemical shift perturbations Δδ_NH_ were quantified relatively to five reference spectra using the following equation:

$$\Delta \delta {\;}_{\mathrm{NH}}={\left(\frac{{\left({\delta^{1}H}_{\mathrm{mix}}-{\delta^{1}H}_{\mathrm{apo}}\right)}^{2}+{\left({\delta^{15}N}_{\mathrm{mix}}-\frac{{\delta^{15}N}_{\mathrm{apo}}}{5}\right)}^{2}}{2}\right)}^{\frac{1}{2}}$$

where δ^1^H_apo_ and δ^15^N_apo_ represent the chemical shifts of the apo-protein and δ^1^H_mix_ and δ^15^N_mix_ the chemical shift of the protein in the presence of the fragments mixture^39^. For every mixture, the averaged Δδ_NHs_ was calculated and the mixtures were then classified using this parameter. The mixtures that could include binders were analysed with the ligand-based experiments. Each 1 D analysis consisted of STD^40^ and WaterLOGSY^41^ experiments in the presence and in the absence of the protein. STD experiments were acquired with 320 scans. Selective saturation of the protein at 0.3 ppm frequency was carried out by a 3 s pulse train (60 Gaussian pulses of 50 ms separated by 1 ms intervals) included in the relaxation delay and a 30 ms spin-lock was used to reduce the broad background, protein signal. Water-LOGSY experiments were performed with a 180° inversion pulse applied to the water signal at ∼4.7 ppm using a Gaussian-shaped selective pulse of 5 ms. Each Water-LOGSY spectrum was acquired with 400 scans and a recovery delay of 2 s between scans. In both experiments, water suppression was achieved by the excitation sculpting pulse scheme^42^. For the ligand-based experiments, the buffer was exchanged against 20 mM PO_4_ (pH 7.4), 150 mM NaCl and 500 μM DTT. The samples contained from 15 to 25 μM of Bcl-X_L_ and 24-fold the protein concentration of each fragment. The same mixtures of 5 fragments analysed in the previous step were used also in the ligand-based screening. For the STD experiments, the amplification factor $f_{\mathrm{STD}},$ was calculated with the following equation:

$$f_{\mathrm{STD}}=\frac{\left(I_{\mathrm{STD}}^{\mathrm{Prot}}-I_{\mathrm{STD}}^{0}\right)}{I_{0}}\frac{{\left[L\right]}_{\mathrm{TOT}}}{{\left[P\right]}_{\mathrm{TOT}}}\cdot 100$$

where $I_{\mathrm{STD}}^{\mathrm{Prot}}$ and $I_{\mathrm{STD}}^{0}$ are peak integrals in the STD experiments with and without protein respectively, $I_{0}$ is the corresponding peak integral in the OFF-resonance STD experiments and ${\left[L\right]}_{\mathrm{TOT}}$ and ${\left[P\right]}_{\mathrm{TOT}}$ are the total concentrations of the ligand and protein respectively. A ligand was considered as a positive in the STD experiment if the amplification factor, calculated with the equation above, had a value of at least 50.

To confirm the binding of the molecules selected from the combined protein- and ligand-based NMR screenings on the mixtures and then also of those deriving from the HT-SuMD based selection on the further 300 fragment screening, ^15 ^N SOFAST-HMQC spectra were acquired on 90 μM ^15 ^N-labelled Bcl-X_L_ in the presence of an eightfold excess (720 μM) of each individual fragment. For the most soluble fragments, a titration was performed with ligand concentrations from 0 to 5.1 mM. Dissociation constants were estimated by monitoring the chemical shift changes as a function of ligand concentration. A grid search was performed using the program OriginPro 2018 b, by varying the values of K_d_ and the chemical shifts of the fully saturated protein and assuming a single-site model. Average values and standard deviations were derived for each K_d_ by monitoring three different peaks in the SOFAST-HMQC spectrum. All NMR spectra were processed and analysed with Bruker TOPSPIN 3.5pl7 (Bruker BioSpin GmbH, Rheinstetten, Germany) and Sparky ^43^.

## 3. Results and discussion

To measure the performance of HT-SuMD in a real drug discovery scenario instead of retrospective validation, we set up a parallel screening comparing NMR and HT-SuMD results. Confident that usually the hit rate in fragment screening for protein–protein interaction usually spans around 2–3%^44^, we selected 100 representative fragments from our in-house library (Library details are available in SI, Dataset-1) including a sizable number of bicyclic aromatic fragments, a scaffold that previously showed an affinity for Bcl-XL target in NMR-screenings^14^^,^^45^.

### 3.1. HT-SuMD screening of 100 fragments library

**Figure 1.**
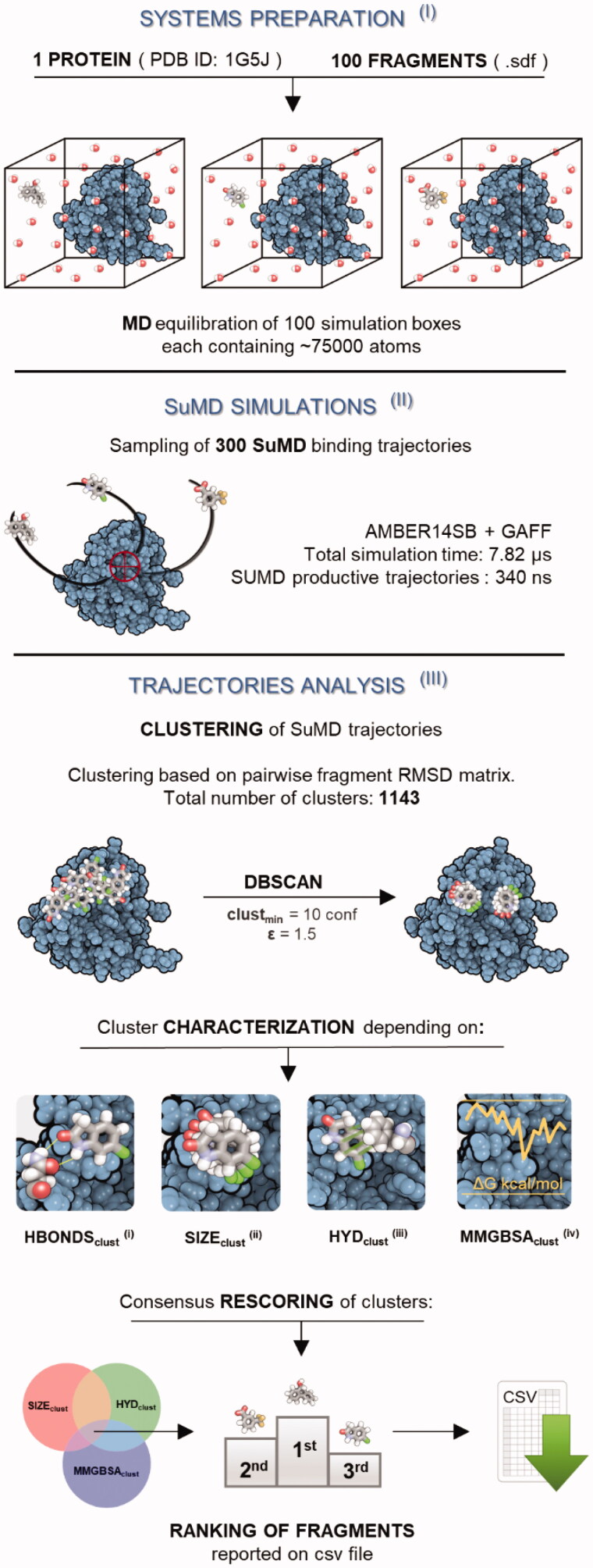
Schematic representation of the computational protocol developed to perform the fragments screening (HT-SuMD). First (I), hundred MD simulation boxes were prepared and equilibrated, placing a single fragment molecule 50 Å away from the Bcl-X_L_ protein binding site (PDB ID: 1G5J). Second (II), 300 SuMD simulations describing putative binding pathways were collected (3 replicas per fragment). Then, the trajectory was geometrically analysed exploiting a density-based clustering algorithm (DBSCAN), to identify highly populated fragment conformations within the protein binding site (III). Each cluster was characterised depending on four geometric/energetic computational descriptors: the presence of a protein–fragment hydrogen bond (i), the cluster size SIZE_clust_ (ii), the average hydrophobic contribution to binding HYD_clust_ (iii), and an energic estimation of binding affinity through MMGBSA_clust_ (iv). A consensus scoring approach was exploited to obtain a ranking of the clusters, and consequently of related fragments.

The implementation of the platform for the HT-SuMD protocol, as reported in Figure 1, entails three main phases: (I) systems preparation and equilibration, (II) SuMD trajectories collection, and (III) analysis of the sampled MD data.HT-SuMD managed the preparation of 100 simulation boxes, each containing a single fragment separated from the protein binding cleft by about 50 Å (step I). To start the in silico simulations from the closest condition to the experimental screening, an NMR solved structure was selected among the Bcl-X_L_ PDB entries available (PDB code: 1G5J)^24^. The small molecules parametrisation was automatically achieved by using the General Amber Force Field. Each simulation box contained roughly 75000 atoms about 96% of which were explicit solvent atoms. To balance the execution time and the sampling of different recognition pathways, we decided to carry out three distinct replicas for each fragment, for a total of 300 putative recognition trajectories (step II). This large amount of simulations leads to a total of 340 ns of SuMD trajectories obtained from a total of 7.82 μs of classical MD (25431 SuMD steps, 300 ps long each). The HT-SuMD production phase required about one week of calculation in a small cluster composed of 20 GPUs. A superposition of all the 300 recognition trajectories is reported in Video S1 (Supplementary Information Video S1). Most of the ligands reached the canonical Bcl-X_L_ binding cleft in a few nanoseconds of productive simulation. It is interesting to note that although the binding site for the supervisor algorithm was set on the centre of the cleft, the fragments have the possibility to explore a vast portion of the protein surface and hence to sample different pockets. This aspect makes SuMD peculiar in comparison to several structure-based techniques focussed on defined binding sites.

To analyse this large number of different trajectories, we develop an automated analysis tool specifically designed for fragments (step III). This phase was crucial because the binding of a fragment is often characterised by weak and transient interactions, making it difficult to uniquely identify a single binding mode. We developed a procedure based on the comparison of well-populated families of molecule conformations characterised by the same geometric binding mode. First, a pairwise RMSD matrix was computed starting from the ligand coordinates along each trajectory, which were then clustered to isolate the densely populated family of conformations from the background noise of the trajectories. Among the various clustering algorithms tested, DBSCAN^46^ proved to be the most performing in discriminating only densely populated clusters. This algorithm has the advantage of not requiring an *a priori* definition of the number of clusters that will be obtained, which are instead created, without any external bias, on the basis of the number of fragment metastable binding sites sampled during the MD simulations.

The 1143 clusters identified were evaluated by considering four computational observables: (i) the presence of a stable hydrogen bond; (ii) the size of the cluster; (iii) a hydrophobic score of the complex conformations, and (iv) the stability of the complex conformation according to the MMGBSA method. A ranking threshold method was used to select the clusters and consequently the related fragments to be considered as potential hits. We decided to monitor the presence of stable hydrogen bonds within each cluster since this interaction plays a pivotal role in anchoring the fragment to the protein binding site (i).

From a comprehensive analysis of three-dimensional structures containing protein–fragment complexes available in the PDB database, it has been recently highlighted that almost all of them were characterised by at least one hydrogen bond mediating the interaction^47^. Similarly, it has been described that hydrogen bonds play a pivotal role in stabilising the complexes since fragment binding is generally enthalpy-drive^48^.

The cluster size, or SIZE_clust_, defined as the number of ligand conformations characterised by a small RMSD value, can be seen as an indirect indicator of the stability of the fragment binding mode associated with the cluster (ii). Each cluster was also scored by the magnitude of the hydrophobic interactions, or HYD_clust_, considering the analysis of the crystallographic complexes retrieved in the RCSB PDB performed by Shaw et al (iii). They highlighted that most fragments bury more than 80% of their total solvent-accessible surface area (SA), with a substantial preference in hiding non-polar, rather than polar, SA.^47^ In our protocol, the hydrophobic effect was indirectly modelled considering the molecular contacts between the fragment and the binding pocket residues, based on the chemical nature of the atom involved (as described in Experimental Section). The computation was performed for each frame belonging to a single cluster and then the mean score was calculated and taken as representative of the entire cluster. Finally, to obtain a rough estimation of the interaction strength from an energetic point of view, all the clustered conformations were subjected to MMGBSA calculation (iv). In this way, the mean energetic value of each cluster, or MMGBSA_clust_ was obtained and the frame with the best energy was picked as the representative complex conformation of the cluster. In summary, the HT-SuMD analysis tool made it possible to characterise all the 1143 clusters based on their size, the presence or absence of H-Bonds, the hydrophobic contribution to binding and their average energetics value.

**Figure 2.**
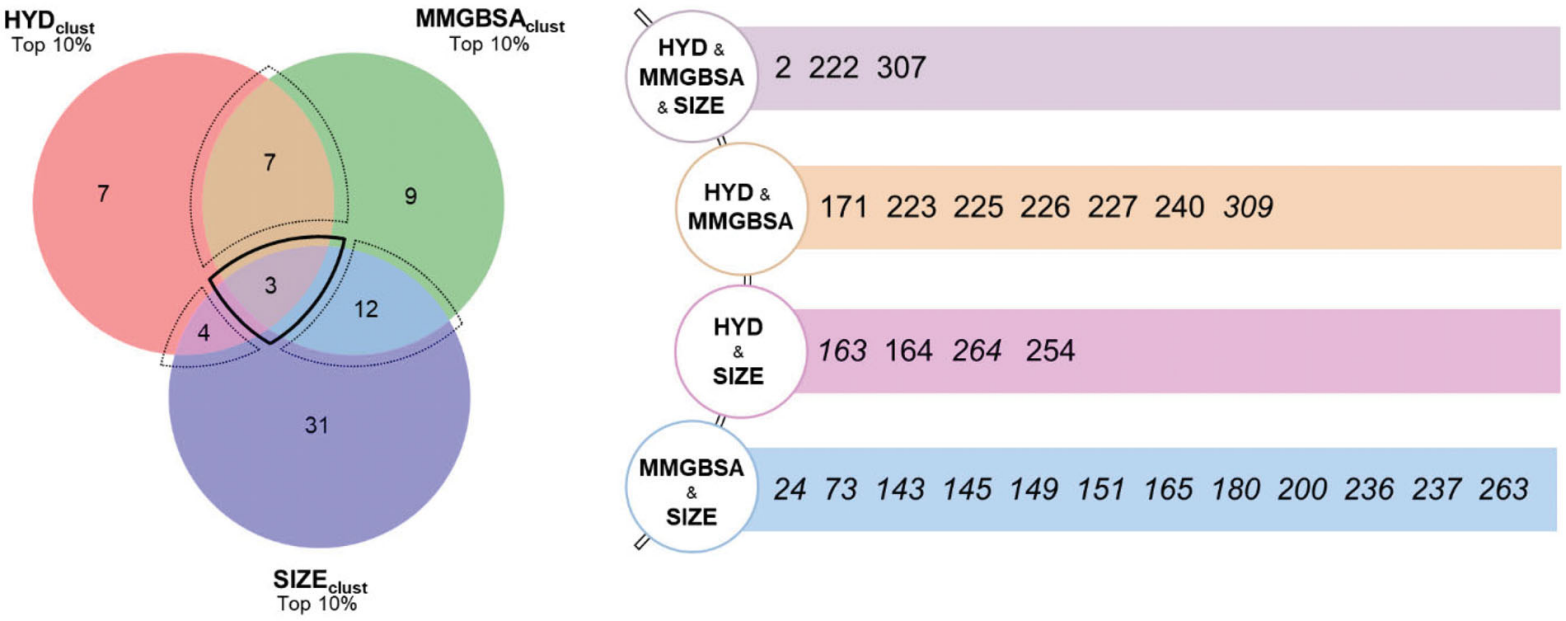
The outcome of the HT-SuMD based fragments screening after consensus scoring of clusters is schematically represented through a Venn diagram, in which the number of non-redundant fragments identified is reported. A bold dashed line contains the molecules showing the greatest convergence of the three computational descriptors, while the thin dashed line delimits the regions of partial computational observables convergence. The areas are proportional to the number of clusters belonging to the region while the number of ligands is not necessarily proportional to the area (i.e. a fragment may be represented by multiple clusters). The ID of the different fragments prioritised by the in silico screening is reported on the right.

To compose a ranking of the clusters, and therefore indirectly also of the fragments, it was necessary to establish a criterion to evaluate and weigh these diverse computational observables. As a first step, in light of the decisive role played by hydrogen bonds, we decided to consider only the clusters characterised by the presence of H-bonds. This entailed an important reduction in the number of clusters to be considered, which dropped from the initial value of 1143 to 681. Then, a consensus approach was developed to sort the remaining clusters. For each of the three descriptors considered, i.e. clusters size (SIZE_clust_), hydrophobic contribution (HYD_clust_), and MMGBSA (MMGBSA_clust_), three independent ranks were built and only the top 10% clusters in each category were kept. Only the clusters, and thus the relative fragments showing consensus among the different scores were taken into consideration. The results are graphically summarised in a Venn diagram (Figure 2) in which the respective intersection areas are proportional to the number of clusters they represent. In the case of multiple clusters associated with the same ligand, only the one with the highest score was retained. As indicated, four different intersections can be distinguished. A first region, delimited by a dashed bold line, corresponds to a maximum convergence among the three observables considered and is populated by 3 fragments (2, 222, and 307). This group contains the most promising fragments according to our analysis protocol and may represent the first choice hit list. In addition, three further intersections are evident, highlighted in Figure 2 by thin dashed lines, which contain fragments for which only two observables converge: HYD_clust_ and SIZE_clust_ (4 fragments); HYD_clust_ and MMGBSA_clust_ (7 fragments); and MMGBSA_clust_ and SIZEclust (12 fragments). In this group, we found fragments with interesting binding modes and with high scores; they could be considered second choice fragments. Finally, we have all the fragments that showed a high score in only one ranking and therefore are less reliable than the previous ones. The scores of such descriptors are reported in SI (SI_HT-SUMD_table.xlsx) for each identified cluster.

### 3.2. NMR-based screening of 100 fragment library

With the aim to validate and optimise the scoring criteria of the in silico approach, the same library of 100 fragments was subjected to an independent NMR based screening. Our protocol relied on both protein- and ligand-based methods, to minimise possible false-positive or false-negative results.

**Figure 3.**
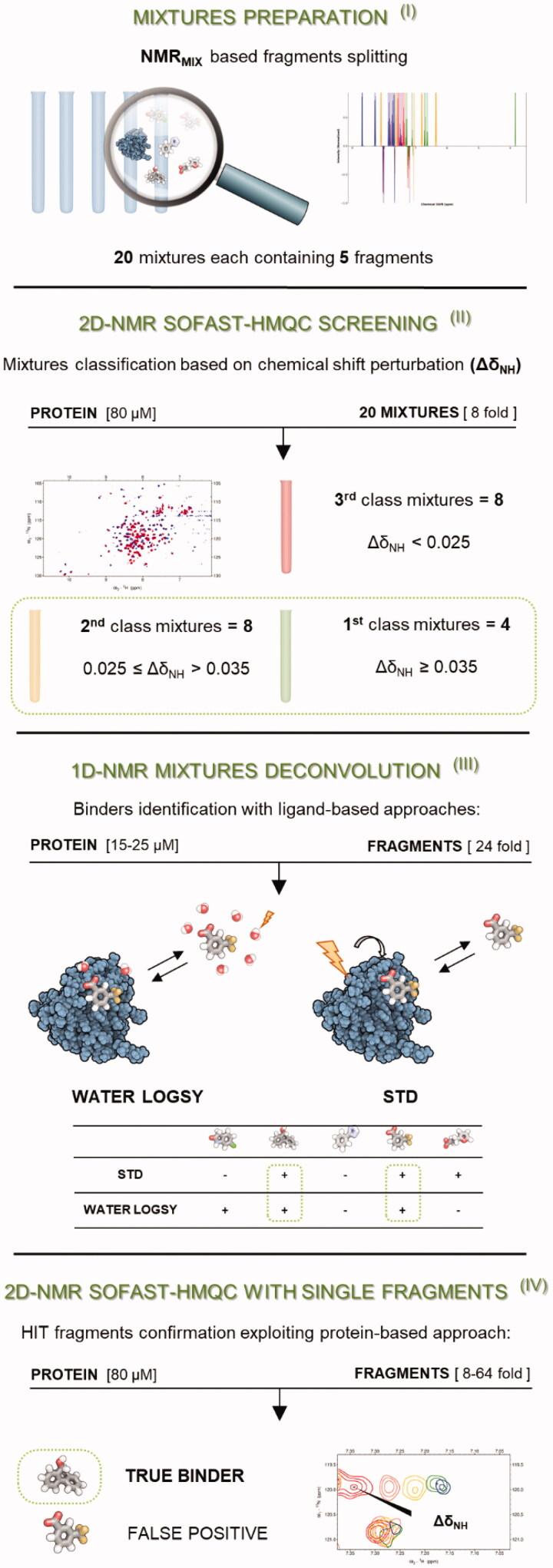
Schematic representation of the cross-validated NMR protocol followed for the initial screening of our small library containing 100 fragments. First (I), the fragments were divided into 20 mixtures each containing 5 molecules, exploiting NMRmix software^37^. Then (II), the protein-based experiment SOFAST-HMQC^39^ was recorded both in the presence and in the absence of the different mixtures, which were classified into three groups depending on their Δδ_NH_ values. Then (III), ligand-based experiments were recorded on the twelve best mixtures to identify the potential binders in every mixture. Finally (IV), the binding of selected molecules was validated exploiting SOFAST-HMQC experiments, collected individually for each fragment.

Briefly, our strategy can be divided into the following steps as reported in Figure 3: (I) fragment mixtures preparation (II) SOFAST-HMQC experiments on 20 mixtures of five molecules each (see methods section) to classify and identify the most promising ones; (III) deconvolution of the most promising mixtures with ligand-based experiments and identification of the potential binding fragments; (IV) final validation of the potential binding fragments with protein-based experiments on the single fragments (when possible, titrations were performed to estimate the affinity constant of the fragment).

The NMR initial screening was based on the more robust protein-based protocol: SOFAST-HMQC experiments were recorded in the absence and in the presence of the fragment mixtures. The choice to start from a protein-based experiment to monitor binding on fragment mixtures relies on the fact that, if the protein is intact and no perturbation of its signals are observed, it is safe to quickly de-validate all ligands of the mixture. With the protein-based approach, we can directly observe if one of the ligands in the mixture harmed the protein. In addition, ^1^H 1 D-spectra were recorded on each sample after the SOFAST-HMQC experiment to verify the integrity and the solubility of the added fragments. It is worth noticing that fast pulsing techniques such as the SOFAST-HMQC, allowed a dramatic decrease in the time required for the acquisition of the 2 D ^1^H-^15^N correlation experiment. As a matter of fact, in our case, the time needed for the SOFAST-HMQC was even shorter than that required to acquire with adequate sensitivity the two ligand-based experiments used in step III. It has also to be considered that our protein of interest, Bcl-X_L_, not only is relatively small and therefore suitable for the protein-based approach but it can be also expressed with good yield, keeping the costs and the time required for the production of the ^15 ^N-labelled relatively limited. Since a high number of mixtures with measurable chemical shift perturbations (CSPs) were identified in the initial screening phase, a careful classification was necessary to select only the mixtures that would potentially include the best binders (step II). To this end, we chose to calculate the parameter Δδ_NH_ only on a selection of 19 non-overlapped residues peaks, located in the protein binding cavity or in its proximity. All other peaks in the spectra were also examined qualitatively to exclude the presence of non-specific binding.

The mixtures were then classified into three classes based on the Δδ_NH_ of the 19 selected peaks (Supplementary Table S1). Four mixtures stood out from the others for exhibiting an average Δδ_NHs_ larger than 0.35 (first class), which suggests a high probability to contain binding fragments. Eight mixtures showed intermediate average Δδ_NHs_ between 0.35 and 0.25 (second class) and, together with the previous ones, were carried to the next step (mixture deconvolution). The remaining mixtures, showing average Δδ_NHs_ below 0.25, were discarded (third class). To identify the interacting molecules in each mixture, the 12 selected mixtures were subjected to Saturation Transfer Difference (STD)^40^ and WaterLOGSY^49^ experiments in the presence and in the absence (control experiment) of the protein (step III). To make this step straightforward, all mixtures had been rationally designed to minimise peak overlap in ligand-based experiments. An overview of all the ligand-based experiments is reported in the Supplementary Material. For 17 fragments (2, 61, 164, 167, 171, 172, 181, 198, 200, 222, 223, 225, 226, 227, 261, 307, 309) the results were unambiguous, and the presence of binding was indicated by both ligand-based experiments. Ten fragments (63, 193, 199, 203, 204, 205, 238, 240, 254, 265) were controversial: water-LOGSY and STD experiments gave opposite results. Supplementary Table S2 summarises the results of the ligand-based experiments. Only those fragments that showed positive results in both experiments were classified as potential binders. In the last step (step IV), the potential binders from the ligand-based experiments were analysed singularly with two-dimensional experiments for the final validation of the interaction. The fragments were tested at the same protein/ligand ratio as in the mixtures to have a direct comparison with the mixture spectra. Among the eight potential binding fragments found in the first class mixtures, six gave significant CSPs in the SOFAST-HMQC spectra and were identified as hits (2, 164, 200, 222, 223, 307). Two fragments (61 and 198) gave very small CSPs and they were classified as false-positives of the ligand-based experiments. All selected fragments in the second class showed significant CSP, except for 181, which was classified as a false positive of the ligand-based experiments.

In our screening, the fragments analysed bind weakly and the protein/ligand ratio is far from saturation. In these conditions, different ligands present in the mixture can bind to the target in a non-competitive way. As a consequence, the chemical shift perturbation induced by different binding fragments present in the mixture should be, at least to a first approximation, additive. We, therefore, verified whether the total CSPs of the mixtures measured in the first step of our protocol was completely explained by the sum of the shifts observed for the single fragments identified as ligands. For 10 out of the 12 selected mixtures, the total measured shift was compatible, within the experimental errors, with the sum of the CSPs induced by the single binding fragments. On the contrary, for the remaining 2 mixtures, the sum of the CSPs of the selected fragments accounted only for about half of what was measured in the mixture. This strongly suggested the presence of false negatives in the ligand-binding experiments. In one of these mixtures two fragments (2 and 198) were classified as hits and one (254) gave conflicting results (binding was indicated only in the STD spectra). Fragment 254 was therefore analysed individually by the SOFAST-HMQC experiment. As a matter of fact, it showed binding to Bcl-X_L_ and it could be identified as a false negative result of the WaterLOGSY experiment. Notably, the sum of the CSPs caused by the single fragments 2, 198, and 254 explained fully the CSPs of the mixture. Finally, in the ligand-based experiments carried on the remaining mixture, only fragment 181 gave positive results in both ligand-based experiments. Nevertheless, in the validation 2 D spectra it did not induce significant CSPs and it was therefore classified as a false positive. In the same mixture, fragments 63 and 240 gave positive results only in one of the ligand-based experiments. Fragment 240 was tested by itself with a 2 D experiment and it induced significant CSPs, which fully explained those measured for the mixture. So, also 240 was a false negative result of the ligand-based experiments. The workflow described above for the NMR screening is very robust as it fulfils all the requirements of the validation cross proposed by Gossert and Jahnke^50^. Binding effects are indeed detected both on the ligands and the protein and the integrity of the fragments and of the target are monitored for each mixture during the screening.

In summary, starting from 100 fragments in 20 mixtures, 12 mixtures were selected for the ligand-based deconvolution. This led to the selection of 17 fragments that gave positive results in both STD and Water-LOGSY experiments and were individually tested by SOFAST-HMQC. The protein-based experiments led to the exclusion of 3 fragments. Finally, comparing the CSPs caused by a mixture and the CSPs caused by the single binding fragments present in the same mixture, 2 false negatives of the ligand-based experiments were identified and classified as binders. In total, 16 out of the initial 100 fragments were selected from this initial screening. A reason for such a wide hit list is the library composition, rich in fragments containing two non-fused rings. As already pointed out by previous studies, this class of molecules could show a higher binding propensity towards Bcl-X_L_^14^^,^^45^.

### 3.3. Comparison between the HT-SuMD and NMR-based screenings

**Figure 4.**
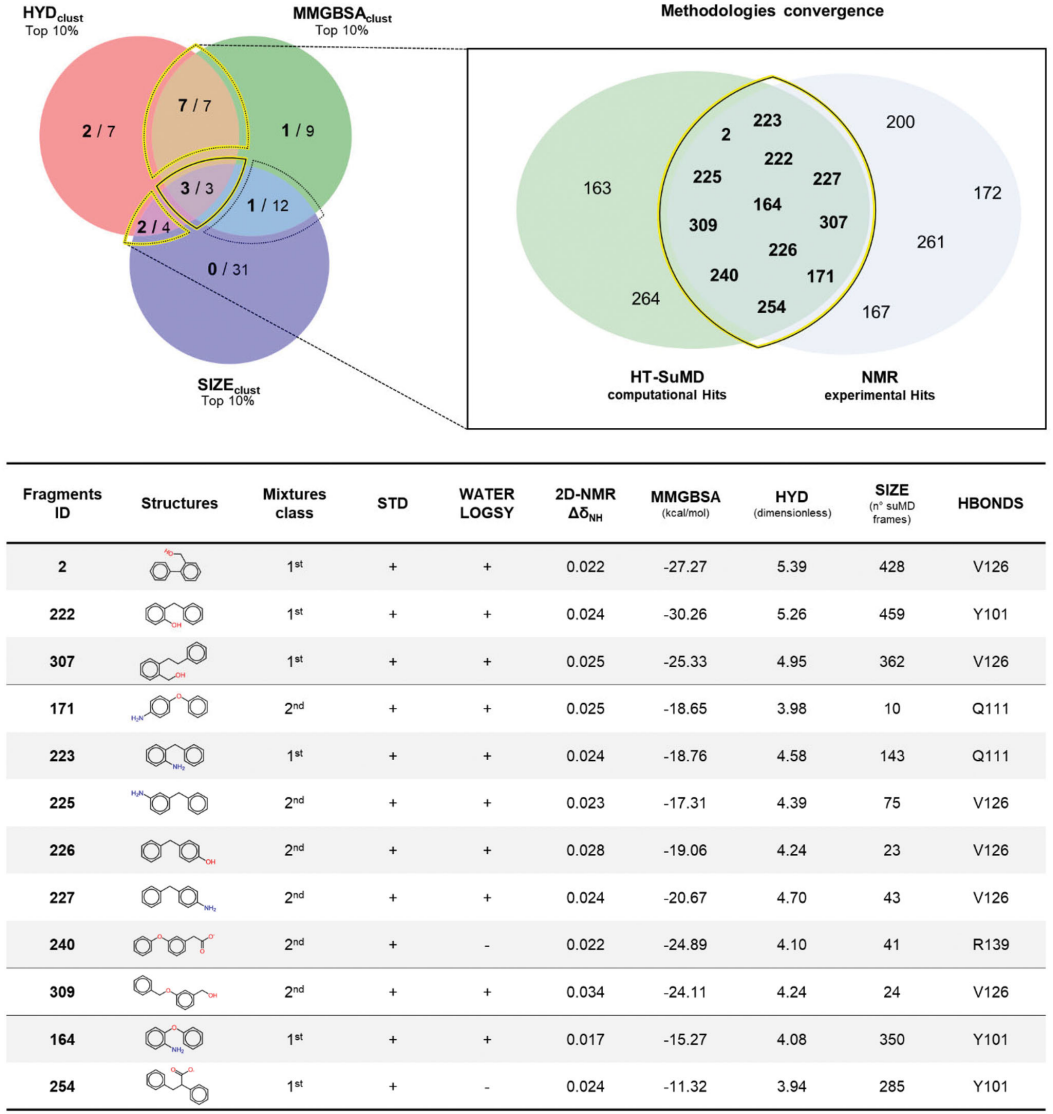
The convergence between the two orthogonal methodologies exploited to perform the fragments screening is depicted through a Venn diagram. On the left panel, the distribution of the 16 active fragments identified using the NMR experimental approach (number in bold font) with respect to the HT-SuMD based clustering (number in normal font) is shown. A yellow line highlights the region where the greatest convergences between the methodologies are found, populated in detail by the molecules belonging to the intersection HYD_clust_ ꓵ SIZE_clust_ ꓵ MMGBSA_clust_, HYD_clust_ ꓵ MMGBSA_clust_ and HYD_clust_ ꓵ SIZE_clust_. On the right panel, a zoom depicting the ID code of the twelve fragments correctly predicted by HT-SuMD protocol is reported; the bottom table summarises, for each of these molecules, the chemical structure, the computational descriptors which drove their choice and the related results of the NMR experiments.

To assess the agreement between the experimental and the in silico screening, a comparison of the respective hits was performed. First, none of the 27 fragments initially excluded because they did not fulfil any of the criteria chosen for the HT-SuMD analysis, proved to be binders in the NMR experiments. For the remaining 73 molecules, the results of the comparison are summarised in Figure 4 through a Venn diagram indicating the overlapping area between the two orthogonal methodologies, along with the chemical structure of the identified fragments and the experimental and computational data supporting the selection. The most remarkable result is the full agreement when focussing on the first-choice hits. All the three top fragments according to HT-SuMD, showing the maximum convergences of computational observables, were also classified as binders in the NMR screening. For two of them, 2 and 222, the Kd was estimated by titrations in protein-based experiments as 1500 ± 500 μM and 1000 ± 400 μM, respectively. From a pragmatical point of view, this result is particularly relevant when considering a drug discovery process in which the maturation of fragments to a lead compound is often focussed only on few but promising fragment hits. A deeper comparison of the two hit lists reveals further interesting points. The second-choice fragments identified using HT-SuMD, i.e. those with only two computational observables in the top 10% score (in the Venn diagram in Figure 2, the three overlapping areas delineated by a thin dashed line) also include fragments for which binding was observed and validated by NMR. Interestingly, the rate of convergence with the NMR data varied considerably among these three subgroups; two subgroups, HYDclust ꓵ SIZEclust and HYDclust ꓵ MMGBSAclust showed an agreement of 50% and 100%, respectively.

The third subgroup, MMGBSAclust ꓵ SIZEclust showed only around 8% agreement. Such peculiar distribution may suggest that some computational descriptors could be more effective in distinguishing true positives. In this case study, HYDclust represents a useful observable, even if this is probably influenced by the topological nature of the pocket hosting the fragment. Bcl-X_L_ has four main hydrophobic pockets in its cleft that are fundamental for the binding of both peptide and small molecule inhibitors. In support of the goodness of the consensus strategy, among the remaining 47 fragments not presenting convergences between computational observables, only 3 were considered true binders in the NMR screening. The vastness of the overlapping area highlights the notable agreement of the computational approach and the NMR-based approach. Retrospectively, if the analysis is focussed on those intersections that showed the greatest convergence between the computational screening and the experimental counterpart, highlighted by a yellow line in Figure 4, 12 fragments out of the 14 predicted by HT-SuMD were correctly identified as binders by NMR (2 false positives). The remaining four fragments, the binding of which was revealed experimentally, were not found in the top of HT-SuMD ranking (4 false negatives, Suppplementary Figure S1). As a result, HT-SuMD showed an accuracy of 94% (as calculated from a confusion matrix) in distinguishing true positive within a large subset of fragment compounds.

### 3.4. Recognition pathway obtained by HT-SUMD for fragment 2

**Figure 5.**
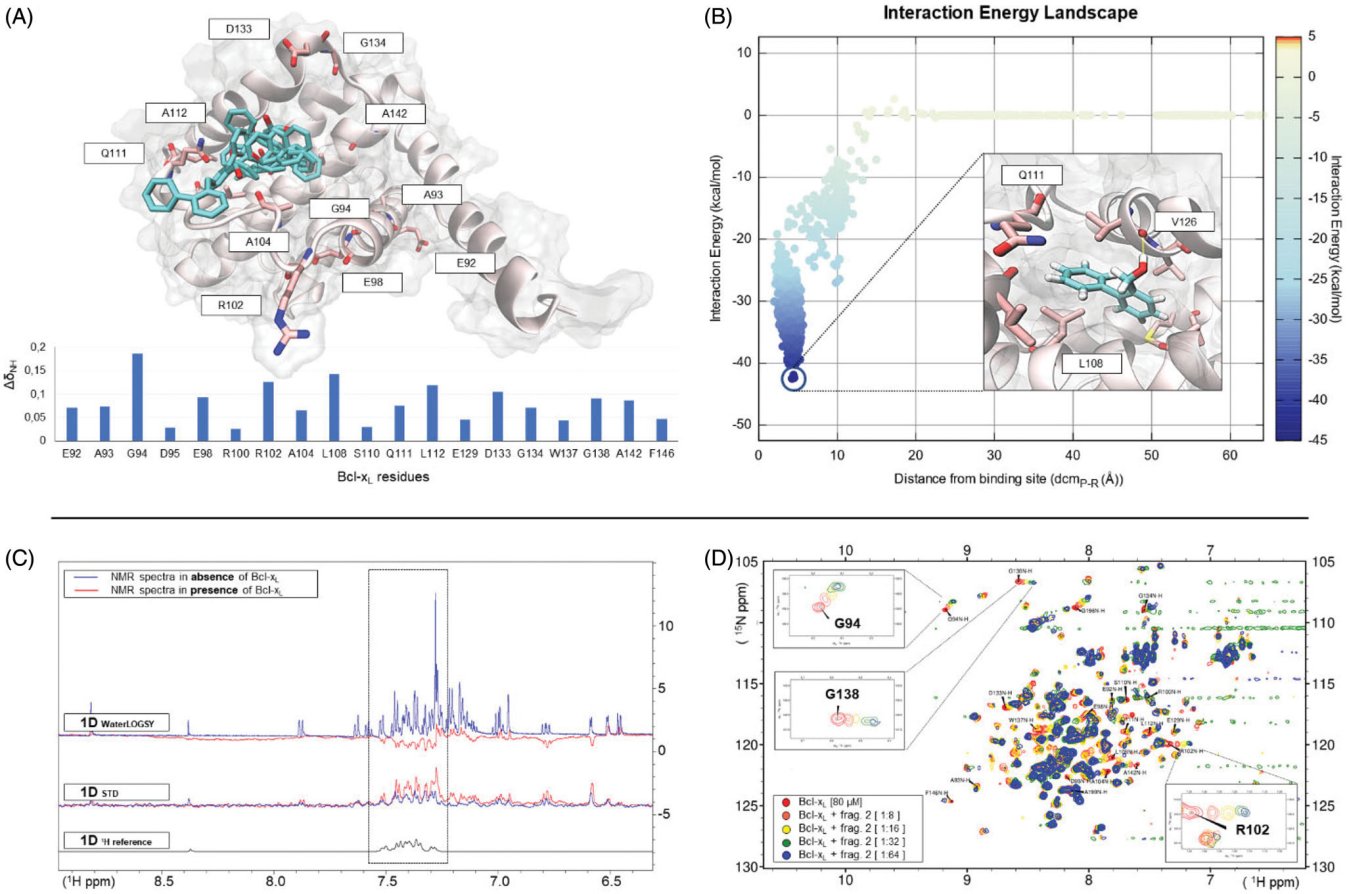
An overview of the computational and experimental information collected relative to fragment 2, one of the best binders identified through the screening campaign, is here summarised. In panel (A), the centroids of each cluster computed starting by the three SuMD simulations sampled are reported within the Bcl-XL binding site. Furthermore, the protein residues that have shown the greatest CSP in the NMR experiments (histogram at the bottom of the panel) are depicted using stick representation, thus confirming the binding site location. Panel (B) reports the Interaction Energy Landscape of one SuMD trajectory describing a putative recognition mechanism, along with the most stable fragment conformation. In panel (C), starting from the top, are reported the Water-LOGSY and STD experiments performed on the entire mixture containing fragment 2, with (in red) and without (in blue) Bcl-X_L_. The reference 1 D NMR experiment of fragment 2 alone is represented in black at the bottom on the same panel. The positive signals observed in the control STD experiment derived from subtraction artefacts, common in STD experiments and caused by instabilities during single FID acquisition, despite on-resonance and off-resonance data have been collected in an interleaved fashion. In the example discussed here, fragment 2 is suggested as a potential binder because it presents negative peaks Water-LOGSY experiment and a significant increase of the STD signals when Bcl-X_L_ is present. Protein-based experiments performed on fragment 2 to validate the binding, are reported on Panel (D), along with a focus on the well-resolved residues peaks, showing a large perturbation during the ligand titration and exploited to estimate the dissociation constant.

Fragment 2 was selected as a representative hit to describe the plethora of information that can be collected through HT-SuMD simulations and that, combined with the experimental data, can guide the fragment optimisation phases. Specifically, in Figure 5(A), all the representative conformations (those with the lowest energy) obtained through cluster analysis of the three different SuMD replicas of fragment 2 are reported. Although the cluster centroids apparently seem very different, a simple comparative analysis reveals a partial overlap among the conformation sampled by the three independent trajectories, suggesting how even different molecular recognition events can converge towards the same metastable sites. It is worth noting that the Bcl-X_L_ residues that have shown the greatest CSP during the 2 D-NMR experiment consistently circumscribe the ensemble of fragment conformations sampled by molecular dynamics, thus confirming the identified binding site. Figure 5(B) reports the SuMD Interaction Energy profile describing the binding event between Bcl-X_L_ and fragment 2, along with the lower energy molecule conformation, which characterises the cluster identified through the consensus ranking procedure. The fragment occupies a hydrophobic pocket at the interface of the protein recognition sites P1 and P2, establishing a stable hydrogen bond interaction with the carbonyl backbone of residue V126 through its hydroxyl moiety. The complete recognition pathway of fragment 2 sampled by means of the SuMD simulation, along with its related geometric and energetic analysis, is summarised in Supplementary Video S2. Fragment 2 binding to Bcl-X_L_ was also experimentally validated, exploiting both a ligand-based (1 D-NMR) and a protein-based (2 D-NMR) approach. As reported in Figure 5(C), the STD spectrum of fragment 2 undergoes a significative increase in peak intensity (mainly in the aromatic region) when acquired in the presence of Bcl-X_L_, with respect to the spectrum of the fragment alone. Similarly, in the waterLOGSY experiment, the presence of negative peaks in the spectrum of the fragment in the presence of Bcl-X_L_ confirms a putative binding to the protein. Finally, the binding epitope of fragment 2 was identified through a protein-based approach (SOFAST-HMQC), and a titration, up to the condition of ligand quasi-saturation, allowed an estimation of its binding affinity in the low millimolar range (K_d_=1500 ± 500 μM).

### 3.5. HT-SuMD screening of 300 fragments library and its validation by NMR

Encouraged by the performance of the in silico protocol and by the agreement with the NMR experimental results, we decided to perform a further screening on 300 different fragments, all retrieved from our in-house library. In this second part of the work, however, the screening was only conducted through a computational approach, using the same HT-SuMD protocol previously described in this manuscript. Only top-ranking fragments were then experimentally NMR-validated, to assess and confirm the ability of the methodology in identifying true binders.

Nine hundred recognition trajectories (300 fragments, 3 replicas each) were thus collected, leading to a total of 960 ns of SuMD productive trajectories sampled, in spite of 22.13 μs of classical MD simulations. The completion of the screening took less than a month of calculation in our small GPUs cluster. This represents, to date, the largest computational screening, entirely based on MD simulations, so far reported in the scientific literature. It is worth noting that exploiting a single GPU driver of the last generation (i.e. NVIDIA Titan V), the HT-SuMD protocol can investigate about 2 molecules/day, in a triplicate way (6 SuMD simulation/day). Considering the high scalability characterising the in silico methodology, with modern GPU clusters exceeding hundreds of devices installed, HT-SuMD makes it possible, in a completely automated way, to screen up to thousands of fragments in a time window that is, after all, quite competitive.

The outcomes of the 900 SuMD trajectories are summarised by Supplementary Video S3, highlighting how the fragment sampling is not strictly confined within the canonical BH3 binding cleft, but it covers a wider portion of the protein surface. Nevertheless, most clusters identified by the geometric analysis performed are preferentially located in a well-known Bcl-X_L_ pocket, named P2, surrounded by the charged residue R139 and other hydrophobic residues. Less explored by fragments during the simulation is the pocket P4, in which only low scored clusters are identified. This is consistent with experimental data reported in the literature, demonstrating that fragments can bind this low-affinity pocket only once the P2 site has been completely saturated by another ligand^51^. This can probably be attributed to the presence of a stationary cluster of water molecules within the P4 site^52^, which are hardly displaced by weak binders, such as fragments.

**Figure 6.**
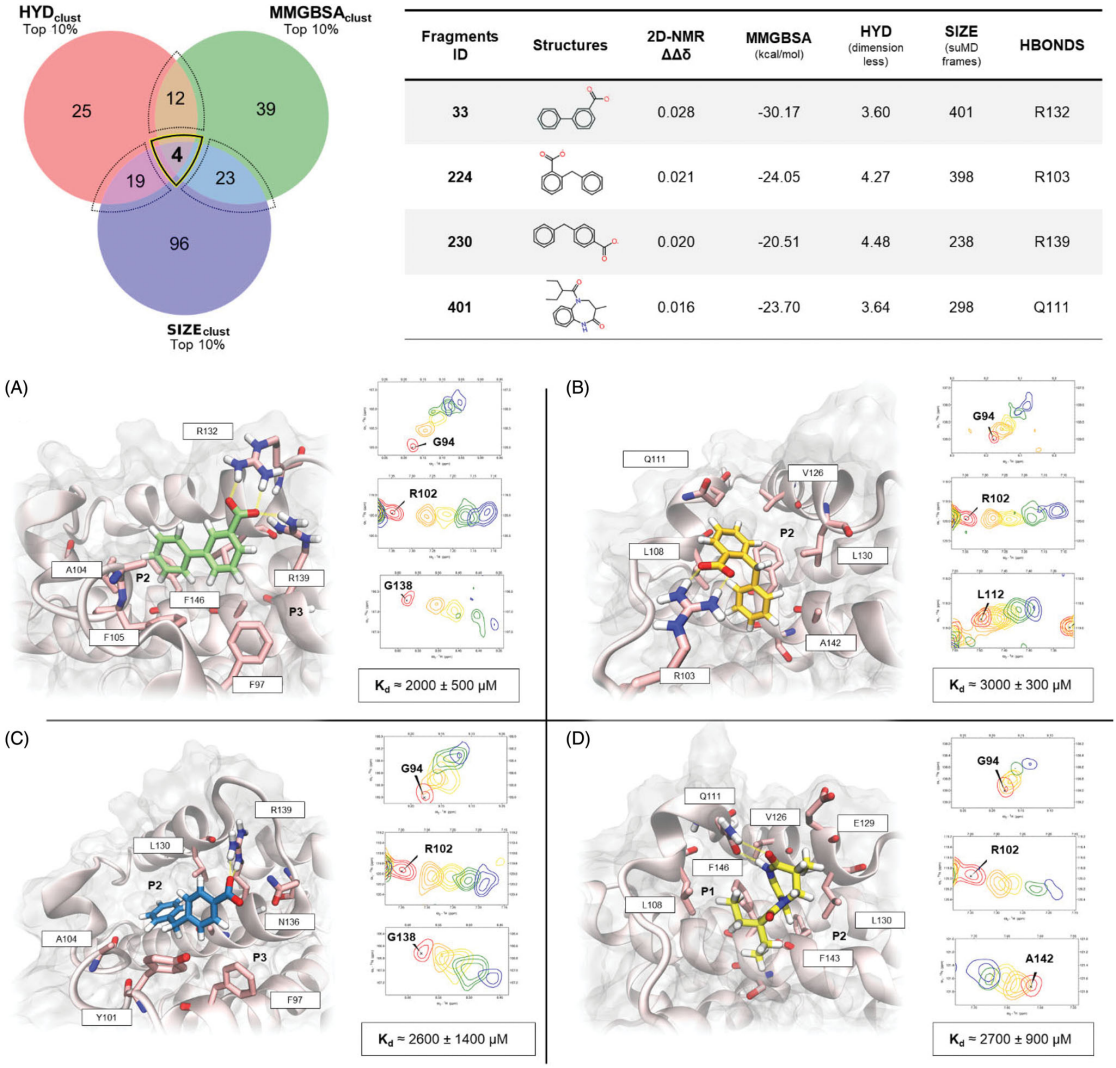
The outcome of the second HT-SuMD campaign of fragments screening, performed on a library composed of 300 molecules, is schematically reported through a Venn diagram after the cluster-based consensus scoring procedure was applied. A bold black and yellow line contain the molecules showing the greatest convergences of the three computational descriptors. The chemical structures of these molecules, which were subsequently experimentally investigated, along with the in silico predicted descriptors for the first-choice class of fragments are reported on the right side of the figure. Panels (A–D) report for each of the four molecules (ID 33, 224, 230, and 401 respectively) the lowest energy conformation sampled; the protein residues involved in fragments binding are rendered as pink liquorice. Protein BCL-X_L_ sub-pocket (P1 to P3) are labelled. Furthermore, for each molecule, the NMR peaks that experimentally showed a large CSP during the ligand titration and that were thus selected to estimate the reported dissociation constant are reported (using the same colorimetric scale previously described in Figure 5).

Since the number of fragments investigated in this second screening was significantly higher than the first one, we pragmatically decided to focus our attention on the first-choice hits fragments, selected applying the same analysis scheme previously described: the mandatory presence of at least one H-bond and concurrent presence of the cluster in the top 10% of the three rankings taken in consideration, respectively MMGBSA_clust_, HYD_clust_, and SIZE_clust_. As highlighted by the Venn diagram depicted in Figure 6, only four fragments, the structures of which are depicted on the right have shown the maximum convergence of computational observables. The selected compounds were hence experimentally investigated through protein-based SOFAST-HMQC protocols and remarkably they all showed the ability to bind Bcl-X_L_, as shown by the Δδ_NH_ chemical shift perturbations. The putative binding mode for all the fragments taken into consideration is reported in Figure 6(A–D) along with the respective K_d_, measured following fragments titration. All the fragments suggested by the in silico protocol experimentally recognise the protein with an estimated K_d_ in the millimolar range, which is quite modest even for a fragment binder; however, the ability of the protocol to identify and prioritise the active compounds within the library is extremely encouraging. Fragments 33, 224, and 230 are all characterised by a bicyclic aromatic scaffold decorated by a negative charged carboxylic acid, which mediates an ionic interaction with one of the arginine residues surrounding the P2 site, as can be seen in Figure 6. Interestingly, fragment 33 has already been identified as a weak Bcl-X_L_ binder in a previous work ^51^, thus confirming the robustness of HT-SuMD. Compound 401, with its 1,5-benzodiazepine scaffold, despite its mild potency, represents an innovative chemotype in view of the design of new potential inhibitors.

## 4. Conclusion

FBLD is an effective strategy to develop new chemical entities able to efficiently bind protein targets. However, fragment screening depends on a reduced number of techniques able to reliably detect weak binding and only NMR and X-ray crystallography provide structural information of binding. Such limitations make it difficult to tackle certain targets^53^. In this respect, computational methods have enormous potential. The calculation of hotspots and the comparison of binding modes are already able to give significant information for the selection and the development of fragment hits.

Here, we have proposed a new application of SuMD that can be used in all those fragment-based screening cases where the structure of the target protein is available. We initially performed in parallel, on the same small library of 100 fragments, an *in silico* screening using HT-SuMD and an experimental one based on a solid, cross-validated NMR approach. In the case of Bcl-X_L_, the protein target object of this study, HT-SuMD has shown an impressive agreement with NMR results, especially in light of the fact that also among different biophysical techniques the convergence could be limited^54^. To our knowledge, this is the first fragment virtual screening based on MD, extensively validated by experimental NMR data. Furthermore, one of the most impressive results is the possibility to explore fragment–target recognition pathways in a reduced time window, three orders of magnitude less than the traditional MD-based approach. The HT-SuMD approach described here was extended to a total of 400 fragments, so far the largest library reported in the literature ever screened with a computational approach entirely based on MD simulations. We hence demonstrated that with this method, it is possible to screen hundreds of fragments in a few weeks, even using a small GPU-cluster. It is worth noting, however, that moving from a small GPUs cluster, like the one used in this study, to a larger infrastructure, would allow shortening the in silico screening, making HT-SuMD extremely convenient also in comparison with experimental approaches. In this respect, we propose that HT-SuMD can be used to efficiently complement experimental methods. First, HT-SuMD represents a valuable tool to prioritise the best fragments binders for experimental screenings. We have indeed shown here that, at least for our target, all four top hits of the in silico screening were weak binders. A wider methodological validation is however necessary, to broaden the applicability domain of HT-SuMD also to biological targets with orthogonal characteristics to those of Bcl-X_L_ (i.e. rigid and hydrophilic binding site).

Also, HT-SuMD can quickly provide precious structural insight into the protein–fragment complex. This information is particularly valuable for targets that are difficult to crystallise and could, therefore, be useful in driving the phase subsequent to the candidate identification, or its maturation towards a lead compound.

The investigation of the association process in HT-SuMD has also several advantages in comparison to methodologies focussed only on the bound state. First, in SuMD simulations, the binding site is fully solvated and the role of the different water molecules in the positioning of every single fragment can be analysed during the recognition process. Secondly, it is possible to explore the adaptability of the protein surface in accommodating different fragments with different biophysical profiles. However, there is still a huge space to improve a robust metric for the selection of novel, diverse and easy-to-grow fragments. Finally, the future of the hybridisation of HT-SuMD with all FBDD approaches will allow one to determine more efficiently which fragment could be most suitable to be transformed into a drug candidate using the most convenient synthetic strategies.

## Supplementary Material

Supplemental Material
