# Supplementary material for: HT-SuMD: making molecular dynamics simulations suitable for fragment-based screening. A comparative study with NMR
Source: J Enzyme Inhib Med Chem. 2020 Oct 28;36(1):1–14. doi: 10.1080/14756366.2020.1838499 (PMC7598995; doi:10.1080/14756366.2020.1838499)
Supplement: Supplemental Material [file IENZ_A_1838499_SM9319.pdf]

SUPPORTING INFORMATION

HT-SuMD: making molecular dynamics simulations suitable for fragment-based screening. A comparative study with NMR.

Francesca Ferrari [b] ‡, Maicol Bissaro [a] ‡, Simone Fabbian [b], Jessica De Almeida Roger[a], Stefano Mammi [b], Stefano Moro [a], Massimo Bellanda [b] *, Mattia Sturlese [a] *

[a] Molecular Modeling Section (MMS), Department of Pharmaceutical and Pharmacological Sciences, University of Padova, via Marzolo 5, 35131 Padova, Italy

[b] Department of Chemical Sciences, University of Padova, via Marzolo 1, 35131 Padova, Italy

List of content

Content	Description
Figure-S1	Structure of outliar fragments
Figure-S2	Overview of the ligand-based NMR experiments
Dataset-1	Smiles strings of the 100 fragments screened in the first round
Dataset-2	Smiles strings of the 300 fragments screened in the second round
Table S1	Mixture Classification according the $\Delta\delta_{NH}$
Table S2	NMR ligand-based deconvolution of the mixtures
Video-S1	Caption of Video-S1. Superposition of 300 recognition trajectories generated by HT-SuMD in the first Virtual Screening (100 fragments).
Video-S2	Caption of Video-S2. Recognition pathway obtained by HT-SuMD of Fragment 2
Video S3	Caption of Video-S3. Superposition of 900 recognition trajectories generated by HT-SuMD in the second Virtual Screening (300 fragments).

Figure S1.

Fragments ID	Structure	STD	WATER LOGSY	2D-NMR $\Delta\delta_{NH}$	MMGBSA (kcal/mol)	HYD (dimensionless)	SIZE (n° suMD frames)	HBonds
**163**	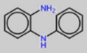	n/a	n/a	0.017*	-13.36	**4.08**	**358**	Q111
**264**	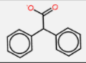	n/a	n/a	0.018*	-11.69	**3.96**	**477**	R103
**200**	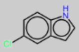	+	+	0.027	**-20.32**	3.77	**795**	F105
**172**	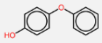	+	+	0.025	-16.50	**4.18**	83	Q111
**261**	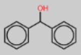	+	+	0.022	-16.05	**4.12**	109	A104
**167**	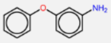	+	+	0.024	**-19.11**	3.83	39	A104

Figure S1 caption

The table summarizes the chemical structure, the computational and experimental NMR descriptors for all the fragment molecules considered as outlier. In detail, four fragments (i.e. 167, 172, 200, 261) were not included by HT-SuMD in the list of putative hits and were independently confirmed by NMR as Bcl-XL binders. It worth noting how fragment 200 was correctly identified by the consensus scoring approach as a potential binder belonging to MMGBSAclust ∩ SIZEclust intersection (Figure 2 and Figure 4). However, being this specific convergence domain rich in false positive, with only one molecule among twelve correctly anticipated as a true binder (i.e. fragment 200), it was decided to exclude this intersection from the hit fragment set. Fragments 167, 172 and 261 instead, all characterized by a bicyclic scaffold, despite the good geometric and energetic indicators, are excluded from the top 10% of the best cluster. The choice of such a stringent cut-off can, therefore, be responsible for the missing identification of the three fragments; however, the use of wider cutoffs could result in increased noise in the selection of true binders molecules. Compound 163 and 164 were instead incorrectly predicted by the HT-SuMD selection protocol as Bcl-XL binders. However, it worth noting how these compounds, belonging to third class mixtures (ΔδNH of the mixtures < 0.025) were therefore discarded from the subsequent screening phase and have never undergone an individual validation phase. The values reported in bolt are the value that are above the theashold (top 10%).

* These values do not refer to the single fragment but refer to the original mixture in which it was contained.

Figure S2

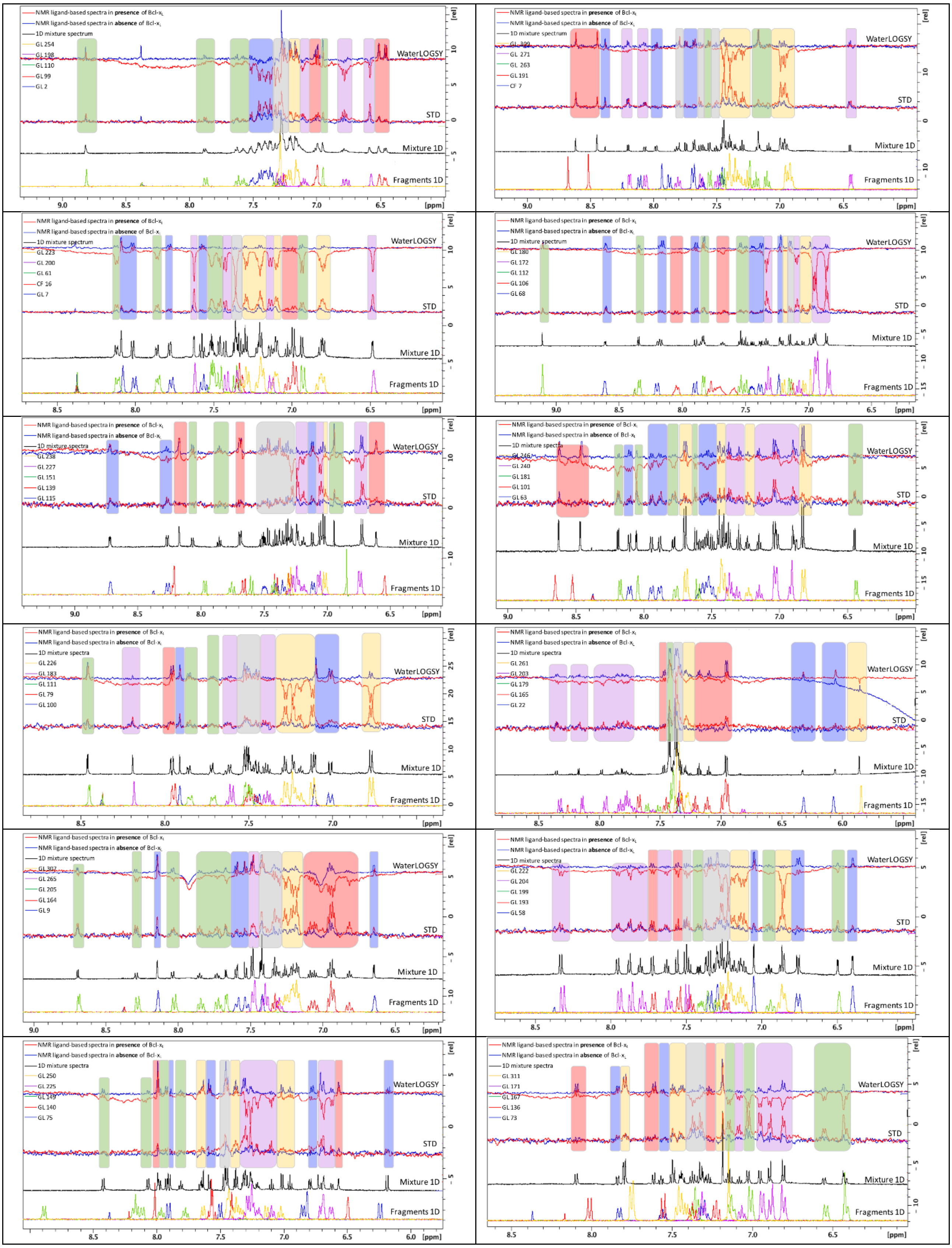
NMR ligand-based spectra in presence of Bcl-xL
NMR ligand-based spectra in absence of Bcl-xL
1D mixture spectrum
WaterLOGSY
STD
Mixture 1D
Fragments 1D
[ppm]
[rel]

Figure S2 caption

Overview of the ligand-based NMR experiments for all the 12 mixtures selected after the initial screening using ^{1}H-^{15}N SOFAST-HMQC experiments. All these mixtures were subjected to Saturation Transfer Difference (STD) and Water-LOGSY experiments in the presence and in the absence (control experiment) of Bcl-X_L and the results are summarized in this Figure. The Water-LOGSY and STD experiments are represented, at the top of each panel in presence (red) and absence (blue) of Bcl-X_L. On the bottom of the panels the 1D NMR of the mixture (black) and the superposition of the 1D experiments acquired on the single fragment and represented with different colours are shown.

Dataset-1. Smiles of the 100 fragments screened in the first round

OCc1c(-c2ccccc2)cccc1	2
FC(F)(F)c1cc(C(=O)[O-])ccc1	7
O=C(N)c1cc2c([nH]cc2)cc1	9
O=C(Nc1ccccc1)c1cnccc1	16
O=C1Nc2c(cccc2)C1	17
[nH]1ncc2c1cccc2	18
O=C([O-])CCc1occc1	22
O=C([O-])CCc1c2c([nH]c1)cccc2	24
O=C([O-])CCc1[nH]c2c(n1)cccc2	25
[nH]1c2ncncc2nc1	26
OCc1[nH]c2c(c1)cccc2	54
Oc1cc2c([nH]cc2)cc1	58
Oc1c2c(ccc1)cccc2	61
OCc1c2c(ccc1)cccc2	63
Oc1cc2c(cc1)cccc2	64
Oc1cc2c(nccc2)cc1	68
Oc1c2ncccc2ccc1	69
O=C1C(=O)Cc2c(O1)cccc2	72
O=C1Oc2c(C(=O)C1)cccc2	73
O=C1Oc2c(cc(O)cc2)C=C1	74
O=C1Oc2c(ccc(O)c2)C=C1	75
c1(-c2ccccc2)nnn[n-]1	79
Nc1cc[n+H]cc1	86
Nc1c2c([nH]cc2)ccc1	99
Nc1cc2c([nH]nc2)cc1	100
O=C1N=C([O-])Nc2nccnc12	101
Nc1nccc2c1cccc2	106
Nc1ncc2c(c1)cccc2	110
Nc1cnc2c(c1)cccc2	111
Nc1c2c(cncc2)ccc1	112
Nc1cc2c(nccc2)cc1	114
Nc1c2ncccc2ccc1	115

O=C1C(N)=Cc2c(O1)cccc2	116
O=C([O-])c1[nH]c2c(c1)cccc2	134
O=C([O-])c1c2c([nH]c1)cccc2	135
O=C([O-])c1n[nH]c2c1cccc2	136
O=C([O-])c1c2c([nH]cc2)ccc1	137
O=C([O-])c1cc2nc[nH]c2cc1	138
O=C([O-])c1cc2c([nH]cc2)cc1	139
O=C([O-])c1cc2[nH]ccc2cc1	140
O=C([O-])c1c2c(ccc1)cccc2	142
O=C([O-])c1nccc2c1cccc2	143
O=C([O-])c1c2c(ncc1)cccc2	144
O=C([O-])c1c2ncccc2ccc1	145
O=C([O-])c1cnc2c(c1)cccc2	148
O=C([O-])c1nc2c(cc1)cccc2	149
O=C([O-])C=1Oc2c(C(=O)C=1)cccc2	151
N(c1c(N)cccc1)c1ccccc1	163
O(c1c(N)cccc1)c1ccccc1	164
O=C([O-])c1c(Oc2ccccc2)cccc1	165
O(c1cc(N)ccc1)c1ccccc1	167
O(c1ccc(N)cc1)c1ccccc1	171
O(c1ccc(O)cc1)c1ccccc1	172
O=C([O-])c1c(-c2ccccc2)cccc1	179
Clc1cc2NC(=O)Cc2cc1	180
Clc1cc2C(=O)C=COc2cc1	181
Fc1cc2C(=O)C=COc2cc1	182
ClC=1C(=O)Oc2c(cccc2)C=1	183
Clc1[nH]c2c(n1)cccc2	190
Clc1ncnc2[nH]cnc12	191
Clc1n[nH]c2c1cccc2	193
Clc1cc2[nH]ccc2cc1	194
Fc1cc2[nH]ccc2cc1	195
Fc1c2nc[nH]c2ccc1	196
Fc1c2c([nH]cc2)ccc1	198

Fc1cc2c([nH]cc2)cc1	199
Clc1cc2c([nH]cc2)cc1	200
Clc1cc2nc[nH]c2cc1	201
Clc1nccc2c1cccc2	203
Clc1nc2c(cc1)cccc2	204
Clc1c2c([n+H]cc1)cccc2	205
Oc1c(Cc2ccccc2)cccc1	222
Nc1c(Cc2ccccc2)cccc1	223
Nc1cc(Cc2ccccc2)ccc1	225
Oc1ccc(Cc2ccccc2)cc1	226
Nc1ccc(Cc2ccccc2)cc1	227
c1(C2CC[N+H2]CC2)ccccc1	231
Nc1ccc(N2CC[N+H2]CC2)cc1	236
Oc1ccc(N2CC[N+H2]CC2)cc1	237
Clc1ccc(N2CC[N+H2]CC2)cc1	238
O=C([O-])Cc1cc(Oc2ccccc2)ccc1	240
O=C(Nc1ccccc1)c1ccc(N)cc1	246
O=C([O-])c1c(OCc2ccccc2)cccc1	250
O=C([O-])C(Cc1ccccc1)c1ccccc1	254
OC(c1ccccc1)c1ccccc1	261
O=C([O-])Cc1c2c([nH]c1)cccc2	263
O=C([O-])C(c1ccccc1)c1ccccc1	264
OC1(c2ccccc2)CC[N+H2]CC1	265
O=C1c2c(OC=C1)cccc2	271
O=C1c2c(O)cccc2OC=C1	272
[N+H3]CC(c1ccccc1)c1ccccc1	276
n1c2c(cnc1)cccc2	305
OCc1c(CCc2ccccc2)cccc1	307
O(Cc1ccccc1)c1cc(CO)ccc1	309
c1(-c2ccccc2)[nH]ccn1	311
c1(-c2ccccc2)[nH]cnc1	312
O=C([O-])c1c(C(=O)c2ccccc2)cc(C(=O)[O-])cc1	350
O(CC[N+H3])c1ccccc1	368

O=C(NCCN1[C@H](C)N=CC1)COCC 410

O(C)c1c(O)ccc2C(C)=CC(=O)Oc12 464

Dataset-2. Smiles of the 300 fragments screened in the second round

O=C(N)C1CCCCC1 1

O=C(N)C1CC[N+H2]CC1 3

FC(F)(F)c1ccc(C(=O)[O-])cc1 10

O=C(NC)C(C)(C)C 11

O=C(N)C1[N+H2]CCC1 12

O=C(N)C1OCCC1 13

O=C(Nc1ccccc1)c1ccccc1 14

Nc1ccc(C(C)(C)C)cc1 15

FC(F)(F)c1cc(N2CC[N+H2]CC2)ccc1 19

O=C([O-])c1ccc(C(C)(C)C)cc1 20

O(C)c1c(O)cccc1 21

O=C([O-])C[N+H]1CCCCC1 23

S(=O)(=O)(N)CCCC(=O)[O-] 27

O=C(N)CCCCC(=O)N 28

O=C([O-])Cc1ccc(-c2ccccc2)cc1 29

Oc1c(O)cccc1 30

Oc1cc(O)ccc1 31

Oc1ccc(O)cc1 32

O=C([O-])c1cc(-c2ccccc2)ccc1 33

OC1CCCCC1 34

Nc1cc2[nH]ccc2cc1 102

Nc1cc2[nH]ncc2cc1 103

Nc1c2[nH]ccc2ccc1 104

Nc1c2c(ccc1)cccc2 105

[N+H3]Cc1c2c(ccc1)cccc2 107

Nc1cc2c(cc1)cccc2 108

Nc1nc2c(cc1)cccc2 109

Nc1c2c(nccc2)ccc1 113

O=C1NC(N)=NC2NC=NC12 117

[N+H2]=C(N)c1ccccc1 118

S(=O)(=O)(N)c1ccccc1 119

S(=O)(=O)(N)c1cc2c(cc1)cccc2 120

O=C1NC(=O)C2N=CNC2N1 121

O=C([O-])c1[nH]ccc1 122

O=C([O-])c1nc[nH]c1 123

O=C([O-])c1cocc1 124

O=C([O-])c1occc1 125

[N+H3]Cc1cc(-c2ccccc2)ccc1126

O=C([O-])c1ncccc1 127

O=C([O-])c1ccncc1 128

O=C([O-])c1cnccc1 129

O=C([O-])C1CCCCC1 130

O=C([O-])C1[N+H2]CCCC1 131

O=C([O-])C1C[N+H2]CCC1 132

O=C([O-])C1CC[N+H2]CC1 133

OCc1ccc(-c2ccccc2)cc1 141

O=C([O-])c1cc2c(cc1)cccc2 146

O=C([O-])c1cc2c(nccc2)cc1 147

O=C([O-])C=1C(=O)c2c(OC=1)cccc2 150

O=C([O-])C=1C(=O)Oc2c(cccc2)C=1 152

O=C(N)c1c(O)cccc1 153

O=C([O-])c1c(O)cccc1 154

O=C([O-])c1c(N)cccc1 155

O=C(N)c1c(N)cccc1 156

O=C([O-])c1cc(N)ccc1 157

O=C([O-])c1cc(O)ccc1 158

O=C([O-])c1ccc(O)cc1 159

O=C(N)c1ccc(O)cc1 160

O=C(N)c1ccc(N)cc1 161

O=C([O-])c1ccc(N)cc1 162

O=C([O-])c1c(Nc2ccccc2)cccc1 166

O(c1cc(O)ccc1)c1ccccc1 168

O=C([O-])c1cc(Oc2ccccc2)ccc1 169

N(c1ccc(N)cc1)c1ccccc1 170

O=C([O-])c1ccc(Oc2ccccc2)cc1 173

Oc1ccc(-c2ccccc2)cc1 174

O=C([O-])c1ccc(-c2ccccc2)cc1 175

Oc1c(-c2ccccc2)cccc1 176

Nc1c(-c2ccccc2)cccc1 177

Fc1c(-c2ccccc2)cccc1 178

[N+H3]Cc1c(-c2ccccc2)cccc1184

Clc1ncccc1 185

Fc1ncccc1 186

Clc1cnccc1 187

Clc1ncccn1 188

ClC1CCCCC1 189

Clc1c2[nH]ccc2ccc1 192

Clc1c2c([nH]cc2)ccc1 197

Fc1c2c(ccc1)cccc2 202

Clc1c2c(nccc2)ccc1 206

Clc1cc2c(nccc2)cc1 207

Clc1c(O)cccc1208

FC(F)(F)c1c(C(=O)[O-])cccc1209

Clc1c(Cl)cccc1Cl 210

O=C([O-])c1c(O)c(O)ccc1 211

O=C([O-])c1c(N)c(O)ccc1 212

Clc1c(Cl)ccc(O)c1 213

O=C([O-])Cc1cc2c(cc1)cccc2214

Clc1c(O)cc(O)cc1 215

Clc1c(N)cc(O)cc1 216

Clc1c(F)cc(O)cc1 217

O=C([O-])c1cc(O)cc(O)c1 218

Fc1cc(F)cc(O)c1 219

Clc1cc(Cl)cc(O)c1 220

Fc1cc(CO)ccc1 221

O=C([O-])c1c(Cc2ccccc2)cccc1 224

Fc1ccc(Oc2ccccc2)cc1 228

Clc1ccc(Oc2ccccc2)cc1 229

O=C([O-])c1ccc(Cc2ccccc2)cc1 230

Fc1c(N2CC[N+H2]CC2)cccc1 232

Oc1c(N2CC[N+H2]CC2)cccc1 233

Oc1cc(N2CC[N+H2]CC2)ccc1 234

Fc1ccc(N2CC[N+H2]CC2)cc1 235

FC(F)(F)c1cc(CO)ccc1 239

OCC(c1ccccc1)c1ccccc1 241

N(Cc1ccccc1)c1ccccc1 242

O(Cc1ccccc1)c1ccccc1 243

Nc1ccc(/C=C/c2ccccc2)cc1 244

O=C(Nc1ccccc1)c1c(O)cccc1 245

O=C(Nc1ccc(N)cc1)c1ccccc1247

O(Cc1ccccc1)c1c(O)cccc1 248

O(Cc1ccccc1)c1c(N)cccc1 249

O(Cc1ccccc1)c1cc(N)ccc1 251

O(Cc1ccccc1)c1ccc(O)cc1 252

O=C([O-])c1ccc(OCc2ccccc2)cc1 253

[N+H3]C(Cc1ccccc1)c1ccccc1 255

N(Cc1ccccc1)c1ncccc1 256

O(Cc1ccccc1)C1=CC(=O)NC=C1 257

O(Cc1ccccc1)c1c(N)nccc1 258

O(c1ccc(C[N+H3])cc1)c1ccccc1 259

O(c1cc(CO)ccc1)c1ccccc1 260

[N+H3]C(c1ccccc1)c1ccccc1 262

N(c1ccccc1)c1ccccc1 266

O=C([O-])CC1CCCCC1 267

Fc1cc2c(NC(=O)C2)cc1 268

Clc1cc2c(NC(=O)C2)cc1 269

Clc1c2NC(=O)Cc2ccc1 270

O=C1Oc2c(cccc2)C=C1	273
O=C([O-])c1c[nH]cc1	274
Clc1[nH]ccn1	275
Clc1c(Cl)nc[nH]1	277
Clc1n(C)cnc1	278
O=C([O-])CC(c1ccccc1)c1ccccc1	279
O=C(N)c1ccccc1	280
Oc1c(N)cccc1	281
Fc1c(O)cccc1	282
Fc1c(N)cccc1	283
Clc1c(S(=O)(=O)N)cccc1	284
S(=O)(=O)(N)c1c(N)cccc1	285
S(=O)(=O)(N)c1c(F)cccc1	286
O=C([O-])c1c(C(=O)N)cccc1	287
O=C([O-])c1c(C(=O)[O-])cccc1	288
Oc1cc(N)ccc1	289
Fc1cc(O)ccc1	290
Fc1cc(N)ccc1	291
Fc1cc(C(=O)[O-])ccc1	292
Clc1cc(C(=O)[O-])ccc1	293
S(=O)(=O)(N)c1cc(N)ccc1	294
O=C([O-])c1cc(C(=O)[O-])ccc1	295
Oc1ccc(N)cc1	296
Fc1ccc(O)cc1	297
Clc1ccc(N)cc1	298
Fc1ccc(C(=O)[O-])cc1	299
Clc1ccc(C(=O)[O-])cc1	300
S(=O)(=O)(N)c1ccc(N)cc1	301
Clc1ccc(S(=O)(=O)N)cc1	302
S(=O)(=O)(N)c1ccc(C(=O)[O-])cc1	303
O=C([O-])c1ccc(C(=O)[O-])cc1	304
OCc1ccc(/C=C/c2ccccc2)cc1	306
O=C([O-])Cc1c(OCc2ccccc2)cccc1	308

O(Cc1ccccc1)c1ccc(CO)cc1 310

OCc1c(O)cccc1 313

O=C([O-])Cc1c(O)cccc1 314

Clc1c(CO)cccc1 315

Clc1c(CC(=O)[O-])cccc1 316

Fc1c(CC(=O)[O-])cccc1 317

Fc1c(CO)cccc1 318

O=C([O-])Cc1c(CC(=O)[O-])cccc1 319

FC(F)(F)c1c(CO)cccc1 320

c1(-c2ccccc2)[nH]c2c(c1)cccc2 321

c1(-c2ccccc2)[nH]c2c(n1)cccc2 322

O=C([O-])Cc1cc(O)ccc1 323

O=C([O-])Cc1cc(N)ccc1 324

Clc1cc(CC(=O)[O-])ccc1 325

Fc1cc(CC(=O)[O-])ccc1 326

Clc1cc(CO)ccc1 327

OCc1cc(CO)ccc1 328

O=C([O-])Cc1cc(CC(=O)[O-])ccc1 329

FC(F)(F)c1cc(CC(=O)[O-])ccc1 330

S(=O)(=O)(N)c1cc(C(F)(F)F)ccc1 331

OCc1ccc(O)cc1 332

O=C([O-])Cc1ccc(O)cc1 333

O=C([O-])Cc1ccc(N)cc1 334

O=C([O-])c1ccc(C[N+H3])cc1335

O=C([O-])c1ccc(CO)cc1 336

S(=O)(=O)(N)c1ccc(C(F)(F)F)cc1 337

FC(F)(F)c1ccc(CO)cc1 338

O=C([O-])c1c(C(=O)[O-])nc[nH]1 339

OCc1ccc(C(C)(C)C)cc1 340

O=C([O-])CC12CC3(NC(=O)C)CC(C1)CC(C3)C2 351

O=C1N2C(=CC=C1)C1C[N+H2]CC(C2)C1 352

O=C([O-])C12C3(C)C(C)(C(C1)CC3)CO2 353

O(C(C)(C)C)C(=O)N1C(C(=O)[O-])CCC1 354

O=C([O-])CCc1c(C)[nH]nc1O 355

O=S1(=O)CC(n2c(C)cc(C)n2)CC1 356

O=C(NC(C)C)NC1(C)CS(=O)(=O)CC1 357

Fc1c(NC(=O)CN2CC(C)OC(C)C2)c(F)ccc1 358

O=C([O-])C1CN(CC=C)C(=O)C1 359

O=C([O-])C12C(O)C(C)(C)C(C1)CC2 360

O=S1(=O)C=CC(O)C1 361

O=C(N)C1CC(=O)NC1 362

O=C([O-])C1C(C(=O)Nc2c(C)ccc(C)c2)CCCC1 363

O=C(N)C1(C)C(C)(C)C(C(=O)[O-])CC1 364

O=C([O-])c1c(C)c2C(O)CCCc2o1 365

O=C1N(C)CCC1O 366

CC1N(c2nnc(N3CC[N+H2]CC3)cc2)CCCC1 367

O=C(N)C1[N+H2]CCCC1 369

OCC12N3C(C)(OC1)CCC3(C)OC2 370

S(=O)(=O)(C)N1C(C(=O)[O-])CCCC1 371

O(C)c1c(OC)cc(c(OC)c1)C1SCC[N+H2]1 372

O=C(OC)C1=C(N)CC(C)S1 373

O=C([O-])CC1N2C(=O)C=C(C(C)(C)C)[N+H]=C2SC1 374

[N+H3]CC1CN(CC[N+H](C)C)CC1 375

O=C(OC)C1CC(=O)NNC1 376

O=C1N(C)CC(C#N)C1 377

O=C1NCC(C(C)C)C1 378

O=C(OC)C1C(=O)NCC1C(C)C 379

O=C([O-])C1NC(=O)CCSC1 380

O=C(N)C1C(C)(C)C1 381

O(C(C)(C)C)C(=O)N1C(C#N)COCC1 382

O(C(C[N+H3])c1sc(C)cc1)C 383

O=C1N2C(C)(NCCC2)CC1 384

O=C(OC)N1CC(C(=O)[O-])CCC1 385

O(CCN1C(=O)C(C)NC1=O)C 386

[N+H2](CC1OCCOC1)C 387

OC(C[N+H3])C1CCCCC1 388

OC(CC[N+H3])c1c(C)oc(C)c1 389

O=C(OC(C)(C)C)CC1[N+H2]CCC1 390

O=C(OC(C)(C)C)NC1CC(O)CCC1 391

S(=O)(=O)(NCC1CN(c2ccccc2)C(=O)C1)CCC 392

O=C(N1CC(C)CC(C)C1)c1c(OC)nn(C)c1 393

O=C(NCC([N+H](C)C)c1cscc1)C(=O)NCCOC 394

O=C(N(C)C1(C)CS(=O)(=O)CC1)c1occc1 395

O=C(NC(COC)C)C(=O)NC1CC1 396

O=C([O-])CC12CC3(O)CC(C1)CC(C3)C2 397

O=C([O-])CC(C)n1c(C)nc(C)n1 398

O=C([O-])C1CS(=O)(=O)CC1 399

O=C([O-])C1NC(=O)C(C)(C)SC1 400

O=C(C(CC)CC)N1c2c(NC(=O)C(C)C1)cccc2 401

O=C(Nc1nn(C)c2NC(=O)CC(C)c12)COCC 402

O=C(NC(Cc1occc1)C)C(C)(C)C 403

O=C(NC(Cc1occc1)C)NC1CC1 404

O=C(NC(Cc1sccc1)C)CC 405

Clc1c(CC(=O)NCC(O)(C)C2CC2)cccc1 406

S(=O)(=O)(NCC(O)(C)c1occc1)CC(C)C 407

O=C(COc1ncccn1)N1CC(C)CCC1 408

O=C(NCC1CS(=O)(=O)CC1)Cc1sccc1 409

O=C(NCC(O)(COC)C)Nc1sccc1 411

O=C(CN1CC[N+H](CC(O)C2CC2)CC1)N1CCCC1 412

O=C(NC1CCCCC1)N1CC(COC)CCC1 413

O=C(C)N1CC(C(=O)NC(CCc2ccccc2)C)C1 414

O=C(C)N1CC(C(=O)NC2C(=O)SCC2)C1 415

S(=O)(=O)(C)N1CC(C(=O)N2CC(C)OC(C)C2)C1 416

Fc1c(C[N+H2]C2CC(O)C(CO)C2)ccc(F)c1 417

O=C(NC1CC(O)C(CO)C1)c1cscc1 418

O=C(Nc1c(C)cccc1)NC1CC(O)CCC1 419

S(=O)(=O)(NC1CC(O)CCC1)c1ccccc1 420

S(CCC(O)(CNC(=O)COc1c(C)cccc1)C)C 421

O=C(OCC)NCCC(O)C1CC1 422

O=C(NCCC(O)C1CC1)C=1C(=O)N(C)C=CC=1 423

O=C(NCC(O)(Cc1occc1)C)NC1CCCCC1 424

Clc1sc(C(=O)NC2CN(C(=O)C2)C2CC2)cc1 425

O=C(OC(C)(C)C)NCC(=O)NC1CN(C(=O)C1)C1CC1 426

O=C(NCCc1onc(C)c1)C1CC=CCC1 427

O=C(NCC#CC[N+H](C(C)C)C(C)C)C1CC=CCC1 428

FC(F)(F)c1oc(C2CN(C(=O)CC)CCC2)nn1 429

O=C(N1C2COCC1CC2)c1cscc1 430

O=C(NCC1(O)c2c(cccc2)CC1)C1CC=CCC1 431

O=C(NCC1(CO)CC1)C1CC=CCC1 432

S(=O)(=O)(NCC([N+H](C)C)c1cc2c(OCC2)cc1)C 433

O=C(NCC(O)c1cocc1)C(C)(C)C 434

O=C(NC(Cc1cocc1)C)COC 435

O=C(N(OC)C)C1N(Cc2sccc2)C(=O)COC1 436

O=C(C(C)(C)C)N1C(C[N+H](C)C)CSCCC1 437

O=C(NCC)N1C(C[N+H]2CCOCC2)CSCCC1 438

O=C(NC(C)(C)C)NCC(O)c1cc(OC)c(OC)cc1 439

O=C(NCCC(O)c1cocc1)COCC 440

O=C(OCC)NCC1(OC)CSCC1 441

S(C)C1CN(C(=O)c2ccc(N(C)C)cc2)CC1 442

S(C)C1CN(C(=O)COc2c(OC)cccc2)CC1 443

S(=O)(=O)(C(C)(C)C)C1CN(C(=O)C2CC=CCC2)CC1 444

O=C(NCC(O)(C)C1CC1)c1c(C)onc1 445

S(CC(O)(CNC(=O)CSC1CCCC1)C)C 446

S(=O)(=O)(NCC(O)C1CCCCC1)c1cn(C)nc1 447

O=C(NCC1OCCC1)c1snc(C)n1 448

O=C(NCc1nn(C)c(C2CC2)c1)C1CC=CCC1 449

S(C)C1CC2N(C(=O)c3c(C)nns3)C(C1)CC2 450

S(C)C1CC2N(C(=O)C3CC=CCC3)C(C1)CC2 451

O=C(Nc1cn(C2COCC2)nc1)C1NC(=O)CC1 452

S(=O)(=O)(N(C)C)Nc1cn(C2COCC2)nc1 453

O=C(NCC(OC)c1c(OC)cccc1)C(C)(C)C 454

S(=O)(=O)(NCC(OC)c1c(C)cccc1)C1CC1 455

O=C(NCC(OC)c1cc(OC)ccc1)C1CC=CCC1 456

Clc1cc(C(OC)(CNC(=O)COCC)C)ccc1 457

O=C(N1CC(Oc2sccn2)C1)C1CC=CCC1 458

S(CC(=O)N1CC(n2nncc2)CC1)C1CCCC1 459

O=C(C(CC)c1ccccc1)N1CCC(n2nncc2)CC1460

Clc1c(C2SCCN(C(=O)COC)CC2)cccc1 461

OC1C(C)(C)CCC1 462

O=C([O-])C1C(C)CCC1 463

Table S1.

**Mixture Number**	**Average $\Delta\delta_{NH}$**	**Mixture Number**	**Average $\Delta\delta_{NH}$**
1	0.016	11	0.030
2	0.016	12	0.016
3	0.035	13	0.017
4	0.027	14	0.025
5	0.040	15	0.038
6	0.028	16	0.039
7	0.014	17	0.025
8	0.028	18	0.022
9	0.018	19	0.030
10	0.028	20	0.018

Table S1 legend: Average $\Delta\delta_{NH}$ of the 20 mixtures of the 100 fragments NMR screening. The first-class mixtures are reported in green, the second-class ones in yellow and the third-class ones in white.

Table S2.

Mixture Number	Fragment	STD	WL
3	2	+	+
	99	-	-
	110	-	-
	198	+	+
	254	+	-
5	7	-	-
	61	+	+
	200	+	+
	223	+	+
	CF16	-	-
15	9	-	-
	164	+	+
	205	-	+
	265	+	-
	307	+	+
16	58	-	-
	193	-	+
	199	-	+
	204	-	+
	222	+	+
4	191	-	-

	263	-	-
	271	-	-
	309	+	+
	cf7	-	-
6	68	-	-
	106	-	-
	112	-	-
	172	+	+
	180	-	-
8	115	-	-
	139	-	-
	151	-	-
	227	+	+
	238	+	-
10	63	-	+
	101	-	-
	181	+	+
	240	+	-
	246	-	-
11	79	-	-
	100	-	-
	111	-	-
	183	-	-

	226	+	+
14	165	-	-
	179	-	-
	203	-	+
	261	+	+
	22	-	-
17	75	-	-
	140	-	-
	149	-	-
	225	+	+
	250	-	-
19	73	-	-
	136	-	-
	167	+	+
	171	+	+
	311	-	-

Table S2 legend: Deconvolution of the first- and second-class mixtures with the ligand-based STD (column STD) and WaterLOGSY (Column WL) experiments. The sign “-” indicates the absence of binding, the sign “+” stands for the presence of binding.

Caption of Video-S1. Superposition of 300 recognition trajectories generated by HT-SuMD in the first Virtual Screening. All the recognition trajectories of the100 fragments of the first screening (3 replicas each) were superposed based on the Ca protein atoms and aligned to the first trajectory frame. Only one protein trajectory is shown for clarity (gray molecular surface).

Caption of Video-S2. Recognition pathway obtained for fragment 2 by HT-SuMD. The best replica according to the analysis protocol was chosen. The video in composed by four synchronized and animated panels that depict the molecular trajectory considering different aspects of the simulation. The time evolution is reported in nanoseconds. In the first panel (upper-left), the molecular

representation of the macromolecular system is shown. The Bcl-X_L molecular surface is reported in light grey while Fragment 2 is rendered using light-green stick representation and by a transparent molecular surface.

In the second panel (upper-right), the distance between the centers of mass of the fragment and Bcl-X_L cleft is reported along the trajectory.

In the third panel (lower-left), the MMGBSA energy profile is reported. The animated red circle highlights the value for the corresponding frame. The trend is depicted by a continuous black line obtained by smoothing the raw data (gray circles) using a Bezier curve procedure.

In the fourth panel (lower-right), the cumulative electrostatic interactions are reported for the 10 Bcl-X_L residues most contacted by the fragment during the whole simulation.

Caption of Video-S3. Superposition of 900 recognition trajectories generated by HT-SuMD in the second Virtual Screening. All the recognition trajectories of the 300 fragments of the second screening (3 replicas each) were superposed based on the Ca protein atoms and aligned to the first trajectory frame. Only one protein trajectory is shown for clarity (gray molecular surface).
